# Structural and mechanistic insights into symmetry conversion in plant GORK K^+^ channel regulation

**DOI:** 10.1093/procel/pwaf067

**Published:** 2025-08-03

**Authors:** Qi-yu Li, Li Qin, Ling-hui Tang, Chun-rui Zhang, Shouguang Huang, Ke Wang, Gao-hua Zhang, Ning-jie Hao, Qian Xiao, Tongxin Niu, Min Su, Rainer Hedrich, Yu-hang Chen

**Affiliations:** Zero-One Innovation Center and Laboratory of Advanced Breeding Technologies, Institute of Genetics and Developmental Biology, Chinese Academy of Sciences, Beijing 100101, China; College of Advanced Agricultural Sciences, University of Chinese Academy of Sciences, Beijing 100049, China; Zero-One Innovation Center and Laboratory of Advanced Breeding Technologies, Institute of Genetics and Developmental Biology, Chinese Academy of Sciences, Beijing 100101, China; College of Advanced Agricultural Sciences, University of Chinese Academy of Sciences, Beijing 100049, China; Zero-One Innovation Center and Laboratory of Advanced Breeding Technologies, Institute of Genetics and Developmental Biology, Chinese Academy of Sciences, Beijing 100101, China; College of Advanced Agricultural Sciences, University of Chinese Academy of Sciences, Beijing 100049, China; Zero-One Innovation Center and Laboratory of Advanced Breeding Technologies, Institute of Genetics and Developmental Biology, Chinese Academy of Sciences, Beijing 100101, China; College of Advanced Agricultural Sciences, University of Chinese Academy of Sciences, Beijing 100049, China; Molecular Plant Physiology and Biophysics, Julius-von-Sachs-Institute, University of Würzburg, Julius-von-Sachs Platz 2, D-97082 Würzburg, Germany; Zero-One Innovation Center and Laboratory of Advanced Breeding Technologies, Institute of Genetics and Developmental Biology, Chinese Academy of Sciences, Beijing 100101, China; College of Advanced Agricultural Sciences, University of Chinese Academy of Sciences, Beijing 100049, China; Zero-One Innovation Center and Laboratory of Advanced Breeding Technologies, Institute of Genetics and Developmental Biology, Chinese Academy of Sciences, Beijing 100101, China; College of Advanced Agricultural Sciences, University of Chinese Academy of Sciences, Beijing 100049, China; Zero-One Innovation Center and Laboratory of Advanced Breeding Technologies, Institute of Genetics and Developmental Biology, Chinese Academy of Sciences, Beijing 100101, China; College of Advanced Agricultural Sciences, University of Chinese Academy of Sciences, Beijing 100049, China; Center for Biological Imaging, Institute of Biophysics, Chinese Academy of Sciences, Beijing 100101, China; Center for Biological Imaging, Institute of Biophysics, Chinese Academy of Sciences, Beijing 100101, China; Zero-One Innovation Center and Laboratory of Advanced Breeding Technologies, Institute of Genetics and Developmental Biology, Chinese Academy of Sciences, Beijing 100101, China; College of Advanced Agricultural Sciences, University of Chinese Academy of Sciences, Beijing 100049, China; Molecular Plant Physiology and Biophysics, Julius-von-Sachs-Institute, University of Würzburg, Julius-von-Sachs Platz 2, D-97082 Würzburg, Germany; Zero-One Innovation Center and Laboratory of Advanced Breeding Technologies, Institute of Genetics and Developmental Biology, Chinese Academy of Sciences, Beijing 100101, China; College of Advanced Agricultural Sciences, University of Chinese Academy of Sciences, Beijing 100049, China

**Keywords:** GORK, symmetry conversion, stomatal signaling, cryo-EM, electrophysiology, guard cell

## Abstract

GORK is a shaker-like potassium channel in plants that contains ankyrin (ANK) repeats. In guard cells, activation of GORK causes K^+^ efflux, reducing turgor pressure and closing stomata. However, how GORK is regulated remains largely elusive. Here, we solved the cryo-EM structure of *Arabidopsis* GORK, revealing an unusual symmetry reduction (from C4 to C2) feature within its tetrameric assembly. This symmetry reduction in GORK channel is driven by ANK dimerization, which disrupts the coupling between transmembrane helices and cytoplasmic domains, thus maintaining GORK in an autoinhibited state. Electrophysiological and structural analyses further confirmed that ANK dimerization inhibits GORK, and its removal restores C4 symmetry, converting GORK to an activatable state. This dynamic switching between C2 and C4 symmetry, mediated by ANK dimerization, presents a GORK target site that guard cells regulate to switch the plant K^+^ channel between inhibited and activatable states, thus controlling stomatal movement in response to environmental stimuli.

## Introduction

Potassium ions are essential for plant growth and development, playing roles in osmoregulation, cell expansion, pH homeostasis, and establishing membrane potential and proton motive force (Maathuis, 2009; Sharma et al., 2013). In *Arabidopsis thaliana*, 71 potassium channels and transporters have been identified, among which 9 belongs to shaker-like channel family (Hedrich, 2012). These channels can be categorized as outward rectifying types, including GORK and SKOR, or inward rectifying types, including KAT1, KAT2, AKT1, and AKT2. They sense changes in membrane potential (depolarization or hyperpolarization), regulate potassium transport, and maintain ionic homeostasis, crucial for plant growth and stress response (Amtmann et al., 2004; Mäser et al., 2001; Sharma et al., 2013; Very and Sentenac, 2003; Wang and Wu, 2010).

GORK, also known as guard cell outward rectifying K^+^ channel, is the only outward-rectifying potassium channel in guard cells. Since its discovery in 2000 (Ache et al., 2000), extensive genetic studies have demonstrated its vital role in regulating stomatal movement (Förster et al., 2019; Kim et al., 2010; Xie et al., 2014). Stomata, formed by paired guard cells, control water loss and CO_2_ uptake in response to environmental stimuli, thereby regulating transpiration and photosynthesis (Ache et al., 2000; Hetherington and Woodward, 2003; Hosy et al., 2003; Lawson and Vialet-Chabrand, 2019; Murata et al., 2015; Schroeder, 2003; Sussmilch et al., 2019). Ion channels in guard cells control turgor pressure by mediating ion flux, which in turn regulates stomatal opening and closing (Hetherington, 2001; Sharma et al., 2013; Very and Sentenac, 2003). KAT1 presents the major guard cell potassium uptake channel, required for increasing turgor pressure and volume increase promoting stomatal opening. In contrast, GORK activation causes potassium efflux, reducing guard cell turgor and volume and in turn stomatal closure (Ache et al., 2000; Hedrich, 2012; Hedrich and Geiger, 2017; Hosy et al., 2003; Kim et al., 2010).

Understanding the structure and function of these channels is critical for unraveling their roles in ion homeostasis and stomatal movement. So far, the structural study of plant KAT1 has provided important insights into its activation and role in potassium influx control (Clark et al., 2020; Li et al., 2020). However, the structure and regulatory mechanisms of GORK remain largely enigmatic. Unlike the inward-rectifying KAT1, the outward-rectifying GORK harbors a unique ankyrin (ANK) repeat domain, whose functional role in plant ion channel regulation remains unexplored.

In this study, we report the cryo-EM structures of *Arabidopsis* GORK (*At*GORK) in both autoinhibited state and activatable state. The full-length *At*GORK^FL^ forms a tetramer with elongated cytoplasmic regulatory domains, with its architecture showing a symmetry reduction from four-fold (C4) to two-fold (C2). Remarkably, ANK from neighboring protomers interacts to form dimers, thus reducing symmetry of the tetramer and rendering GORK in an autoinhibited state. Electrophysiological studies show that mutations or truncations disrupting ANK interactions convert GORK into an activatable state. Further structural analysis of ANK-truncated *At*GORK^623^ and *At*GORK^510^ reveals that the cytoplasmic regulatory domains become highly dynamic, while strict C4 symmetry is restored in the tetrameric assembly.

Thus, our study demonstrates that ANK acts as a molecular switch for symmetry conversion (between C2 and C4), regulating GORK conformational transitions between autoinhibited and activatable states. This research provides the first comprehensive elucidation of the biological role of ANK in GORK, offering new insights into how GORK mediates stomatal closure in response to environmental signals. Furthermore, our findings broaden the understanding of ion channel regulation, particularly how symmetry changes in structural assembly regulate ion channel activity.

## Results

### Cryo-EM structure determination of the *Arabidopsis* GORK

To elucidate the structure of the GORK, we prepared *Arabidopsis* GORK (fused with an N-terminal GFP tag) from HEK293 cells and conducted single particle cryo-EM analysis. After solubilization in 1.0% n-Dodecyl-B-D-Maltoside (DDM) and 0.02% cholesteryl hemisuccinate (CHS), the *At*GORK proteins underwent purification through strep-tactin affinity chromatography, GFP removal via TEV enzyme cleavage, and subsequent gel-filtration chromatography. The final solution contained 0.006% detergent GDN, 150 mmol/L NaCl, and 20 mmol/L HEPES-Na pH 8.0. The most monodisperse peak fractions were pooled and concentrated to approximately 3.5 mg/mL for cryo-EM grid preparation.

We determined the cryo-EM structures of the full-length *At*GORK (*At*GORK^FL^, residues 1–820) in an autoinhibited state, along with two ANK-truncated versions (*At*GORK^623^, residues 1–623; *At*GORK^510^, residues 1–510) (Table S1). Tetrameric reconstructions of *At*GORK were obtained in two conformational states at resolutions of 3.4 Å (*At*GORK^FL1^) and 4.3 Å (*At*GORK^FL2^), allowing *de novo* modeling of 662 of 820 amino acids (residues 58–720) for each protomer, while the remaining regions were not resolved due to their intrinsic flexibility (Fig. S1). For the truncated *At*GORK^510^ and *At*GORK^623^, cryo-EM maps at 3.4 Å and 3.2 Å resolution, respectively, were obtained. Both maps showed high-quality density in their transmembrane domain, allowing accurate modeling, while the flexible cytosolic domains remained unmodeled due to their invisibility (Fig. S2). Structure-based sequence alignment for 24 representative GORKs from both monocots and dicots is shown in Fig. S3.

### Architecture of *Arabidopsis* GORK in symmetry-reduced conformation

**Figure 1.**
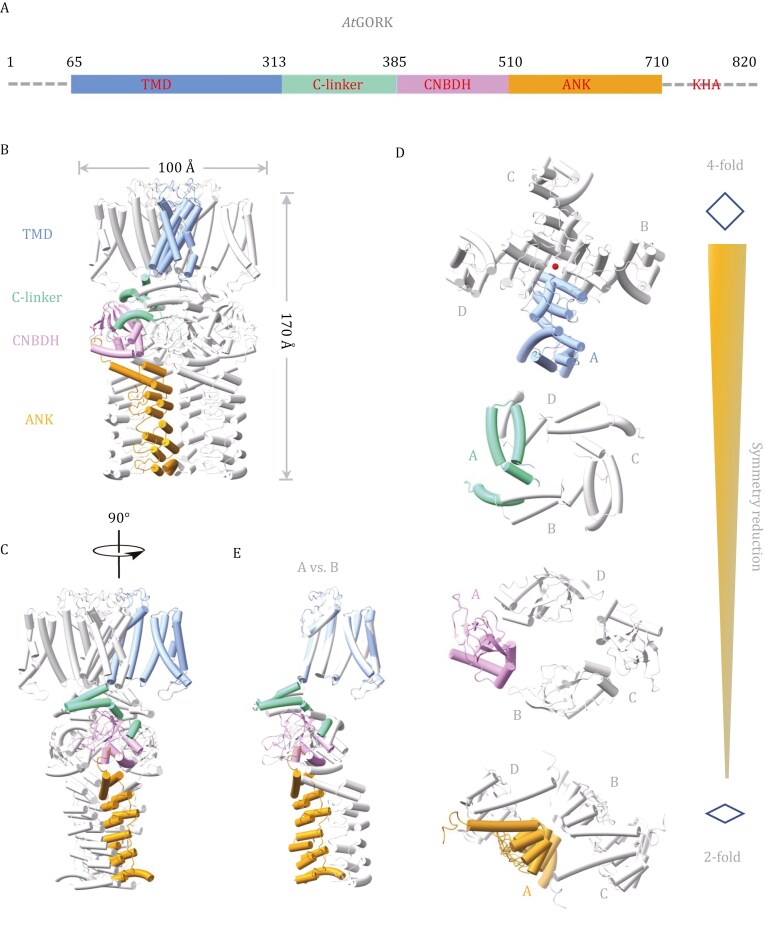
Architecture of the *Arabidopsis* GORK. (A) Schematic diagram of the domain architecture in Arabidopsis GORK (*At*GORK). The transmembrane domain (TMD, blue), the C-linker domain (C-linker, cyan), the CNBDH (purple), and the Ankyrin domain (ANK, orange) are shown. (B and C) The cartoon drawing of the *At*GORK tetramer. One protomer is colored as indicated in (A), and the other protomers are colored in gray. (D) Symmetry reduction (from C4 to C2) within the tetrameric assembly. This represents a unique structural feature of the GORK family and likely serves as a unique mechanism for channel regulation. The current *At*GORK structure likely represents an autoinhibited, preactivation state. Different protomers are indicated with letters A–D. (E) Structural superimposition of protomer A with its neighboring protomer B.

*Arabidopsis* GORK (*At*GORK) forms a homo-tetrameric structure around a central pore, with a dimension of 110 Å (length) × 110 Å (width) × 170 Å (height) (Fig. 1A–C). Each protomer has an N-terminal transmembrane domain (TMD, residues 58–313, containing helices S1–S6), followed by three cytoplasmic domains: the C-linker (residues 314–365), cyclic nucleotide-binding domain homolog (CNBDH) (residues 366–510), and ANK (residues 511–724). The segment preceding the TMD (residues 1–57) and the last portion (residues 725–824, containing a putative KHA domain enriched in hydrophobic and acidic residues) are not visible in the structure, likely due to their intrinsic flexibility or the flexibility of the connecting regions.

The *At*GORK structure, unlike other tetrameric ion channels such as KAT1, exhibits a unique architectural assembly (Clark et al., 2020). Notably, the symmetry in the TMD portion adopts a typical four-fold (C4), but reduces to a two-fold (C2) in their following cytoplasmic domains, thus leading to a deformation within the tetrameric channel (Fig. 1D). Superimposition of its protomer reveals distinct conformations, with notable rotations and displacements occurred in the C-linker, CNBDH, and ANK domains (Fig. 1E). This symmetry reduction within the tetrameric assembly appears to arise from ANK dimerization (will discuss later). To the best of our knowledge, this symmetry reduction in channel assembly is exceptionally rare and leads to deformation of the tetrameric channel. This represents a unique structural feature of the GORK family and likely serves as a unique mechanism for channel regulation. Thus, the current *At*GORK structure likely represents an autoinhibited, preactivation state.

**Figure 2.**
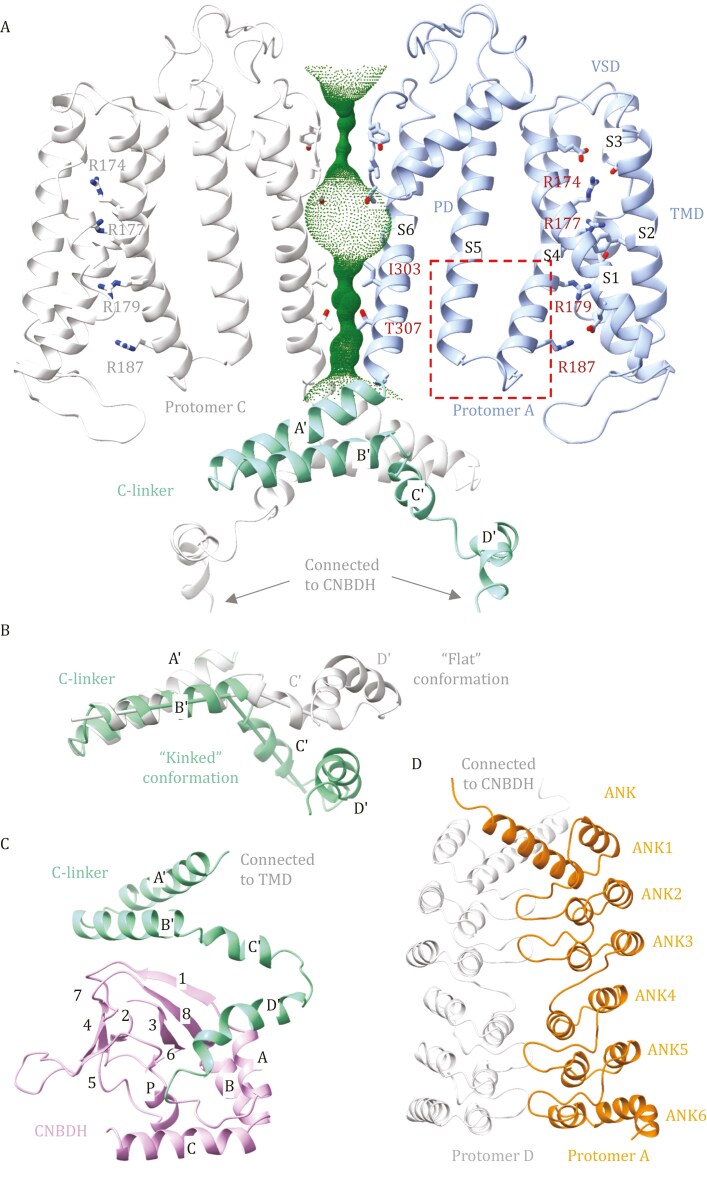
Structural domains of ***At***GORK: TMD, C-linker, CNBDH, and ANK. (A) Ribbons drawing of the TMD/C-linker portion, with two protomers (protomer A and protomer C) are shown, colored and oriented as in Fig. 1C. The VSD is connected to the PD through a short linker, resulting in a “non-domain-swapped” configuration. The pore-lining surface was computed by the program HOLE and drawn into a cartoon model of the GORK channel pore in a closed conformation. Critical residues in VSD (R174, R177, R179, and R187), selectivity filter (^271^TVGYG^275^), and the narrowest constrictions (I303 and T307) are shown in sticks. The S4 helix adopts a resting “up” conformation, similar to that in plant inward rectifying KAT1 and AKT1. (B) Structural comparison of the C-linker of protomer A (“flat” conformation) with protomer B (“kinked” conformation). The “flat” conformation of the C-linker actually disrupts the formation of a C4 symmetrical gating ring, resulting in decoupling between the transmembrane helices with the cytoplasmic domains, ultimately affecting channel gating. (C) Ribbons drawing of the C-linker/CNBDH portion. (D) Ribbons drawing of the dimeric ANK portion. ANKs from two neighboring protomers tightly interact, creating a buried area of ~1,500 Å^2^. The first three and the last three repeats are tandem units. Repeats 1–2/4–5 are canonical α-helix/α-helix/β-hairpin motifs, while repeat 3/6 lacks the β-hairpin. The presence of the ANK domain and its dimerization are unique features of the GORK family. ANK dimerization leads to symmetry reduction in the tetrameric channel. This, in turn, affects the coupling between the transmembrane helices and cytoplasmic domains, ultimately holding the ion channel in an autoinhibited, preactivation state.

The TMD consists of a voltage-sensing domain (VSD, helices S1–S4) and a pore-forming domain (PD, helices S5 and S6) (Fig 2A). The VSD is connected to the PD through a short linker, resulting in a “non-domain-swapped” configuration where the VSD and PD of the same subunit form a cohesive bundle. This differs from the canonical domain-swapped architecture in Shaker-like (Long et al., 2005) and Na_V_ channels (Payandeh et al., 2011), where the VSD and PD are separated by a long S4–S5 linker. The P-loop segment of *At*GORK contains a highly conserved motif (^271^TVGYG^275^), similar to that of bacterial KcsA (Zhou et al., 2001), mammalian HCN (Lee and MacKinnon, 2017), and plant outward-rectifying SKOR (Li et al., 2023) and inward rectifying KAT1 (Clark et al., 2020) and AKT1 (Lu et al., 2022). Structural comparisons reveal that they form a similar K^+^ selectivity filter (Fig. S4A). Analysis using the HOLE (Smart et al., 1996) suggests that the *At*GORK adopts a closed conformation. Below the selectivity filter lies the pore gate region consists of residues on the S6 helix, with I303 and T307 creating the narrowest constrictions (radius of approximately 1 Å). The S4 helix contains conserved positively charged residues (R174, R177, R179, and R187), and adopts a resting “up” conformation, similar to that in plant inward rectifying KAT1 (Clark et al., 2020) and AKT1 (Lu et al., 2022) (Fig. S4B).

After the TMD, the C-linker is positioned next to the helix bundle crossing, connecting the transmembrane helices to the cytoplasmic domains. It comprises four helices (A′–D′), forming two “helix-turn-helix” motifs. These helices further assemble into a gating ring architecture within the tetrameric channel through “elbow-on-shoulder” inter-subunit interactions, where helix A′-turn-helix B′ (elbow) of one protomer interacts with helix C′-turn-helix D′ (shoulder) of its neighboring protomer (Flynn et al., 2007) (Fig. 1C). Due to the reduced symmetry within the tetramer, the C-linkers exhibits distinct conformations between adjacent protomers, either “flat” or “kinked” (Fig. 2B). Consequently, the C-linker in *At*GORK deviates from C4 symmetry, and the presence of the “flat” conformation disrupts the formation of a C4 symmetrical gating ring, leading to decoupling between the transmembrane helices and the cytoplasmic domains, ultimately affecting channel gating.

The CNBDH domain, connected to the C-linker, shares approximately 20% sequence identity with canonical cyclic nucleotide-binding domains (CNBDs) (Fig. S5A), which play crucial roles in regulating channels, kinases and transcription factors upon binding to secondary messengers (cAMP or cGMP). Despite low sequence homology, the CNBDH shares a similar structural fold to those of the CNBDs (Fig. S5B). The CNBDH of GORK consists of four short α-helices (A, P, B, and C) and an eight-stranded β-roll (β1–β8) (Fig. 2C). Helix A is located before the β-roll, Helix P is inserted within the β-roll (between β6 and β7), and Helices B and C are positioned afterward. Helices A and B interact in an antiparallel manner, and the putative phosphate-binding cassette comprises β6, Helix P, and β7.

Although the CNBDH shares overall structural similarity with animal CNBDs, there are notable differences (Fig. S5B). GORK lacks conserved residues for cAMP binding, such as R561 in 7LFX or R620 in 2PTM, which is replaced by Q465. More strikingly, alanine at the binding pocket entrance (A563 in 7lfx (Xue et al., 2021), or A622 in 2ptm (Flynn et al., 2007) is replaced by bulky phenylalanine (F467 in *At*GORK), likely blocking nucleotide access. Helix C also adopts a closed conformation, with its adjacent loop obstructing the binding site entrance, suggesting that large conformational changes would be required for cyclic nucleotide binding.

Following the CNBDH, six ankyrin repeats fold into a single ANK domain, forming a slightly curved solenoid structure (Fig. 2D). The first three repeats (ANK1–3) and the last three repeats (ANK4–6) are tandem units with ~34% sequence identity. In each tandem unit, two repeats are canonical, consisting of a pair of antiparallel α-helices and an intervening “finger” loop, while the third is a degenerate repeat lacking the “finger” loop. Remarkably, ANK repeats from two neighboring protomers form a stable dimer through specific interactions mediated by their protruding loops, creating a buried area of ~1,500 Å^2^. This dimeric interaction is specific, corroborated by another structure of the full-length of *At*GORK^FL2^, which retains similar dimeric units despite of variation in their tetrameric conformations (Fig. S1E). A similar ANK dimerization pattern has also been observed in SKOR (Fig. S1F) (Li et al., 2023), suggesting that this dimerization is a common structural feature in outward rectifying plant ANK-containing K^+^ channels.

As a result of ANK dimerization, positional deviations and conformational changes are induced in the adjacent CNBDH and C-linker, leading to a reduction in symmetry within the tetrameric channel. This reduction affects the communication and coupling between cytoplasmic domains and transmembrane helices, ultimately maintaining GORK in an autoinhibited, preactivation state. The structures of GORK and SKOR with reduced symmetry provide crucial insights into how the ANK domain regulates these channels, maintaining them in an autoinhibited conformation under resting conditions. This is supported by the observation of basal activity of full-length *At*GORK.

### ANK dimerization maintains GORK channel in an autoinhibited state

**Figure 3.**
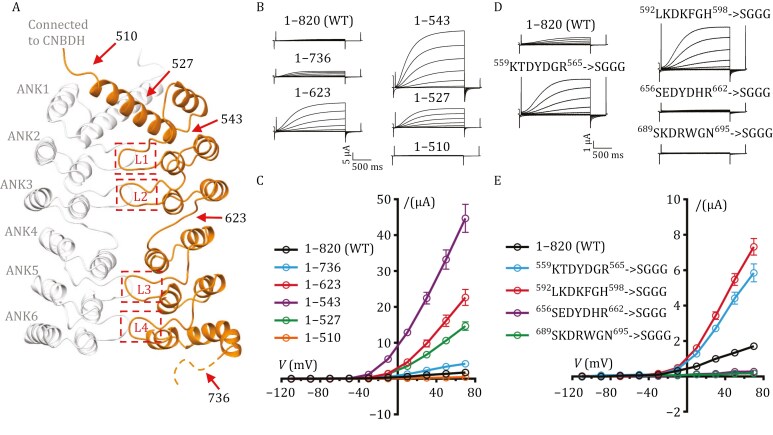
ANK plays an inhibitory role in regulating *At*GORK. (A) Ribbons drawing of the ANK dimeric domain, with truncated points or mutated loops are highlighted with red arrows or in red boxes. (B and C) The C-terminal truncation of *At*GORK, with representative current traces in (B) and steady-state current–voltage (*I*–*V*) relations in (C). Truncating half ANK domain (Δ624–820, residues 1–623) or the entire ANK domain (Δ544–820, residues 1–543) significantly increased currents. However, a further truncation (Δ511–820, residues 1–510) disrupted the channel activity. (D and E) The loop-substitution in ANK of *At*GORK, with representative current traces in (D) and steady-state current–voltage (*I*–*V*) relations in (E). Substitution of loops between repeats 1/2 and 2/3 with “SGGG” significantly enhanced GORK activity. However, similar replacement of the loops between repeats 4/5 and 5/6 impaired GORK function. Data are mean ± SEM, *n* ≥ 8.

The ANK repeats and their dimeric interactions are distinctive features of the GORK channel, playing a key role in reducing symmetry within its tetrameric assembly. To explore their function, we conducted trunctional analysis of the ankyrin repeats in *At*GORK expressed in *Xenopus oocytes* and inspected via TEVC electrophysiology (Fig. 3A). Full-length *At*GORK produced basal currents, indicating that a small fraction of these channels was in an activatable state. Removing the KHA domain while retaining the intact ANK domain in *At*GORK (Δ737–820, residues 1–736) led to a slight increase in oocytes currents, whereas deleting the last three C-terminal ANK repeats (Δ624–820, residues 1–623) significantly enhanced current amplitude—reaching 13.3-fold that of the full-length channel at +70 mV (Fig. 3B and 3C). However, removing all six ANK repeats produced more complex outcomes depending on the truncation site: Δ544–820 (residues 1–543) further increased current amplitude to approximately 26.3-fold that of the wild-type full-length channel, while Δ528–820 (residues 1–527) decreased current amplitude, and Δ511–820 (residues 1–510) nearly abolished the currents (Fig. 3B and 3C).

To rule out the possibility that these changes in current were due to trafficking defects, we fused a GFP tag to the N-terminus of these ANK truncation mutants and observed that their surface expression levels were comparable to that of the wild-type channel (Fig. S6A and S6B). Therefore, the enhanced currents in the truncation mutants are primarily due to the disruption of ANK dimerization, which in turn releases *At*GORK from its autoinhibited state. These findings indicate that ANK dimerization is responsible for maintaining the autoinhibited state of *At*GORK, and the channel becomes activatable upon autoinhibition release. Moreover, the connecting helix from the first ANK repeat (residues 520–543), which is directly connected to the CNBDH, is essential for the transition of *At*GORK from the autoinhibited to the activated state.

The dimerization of ANK is driven by hydrophobic interactions mediated by the hairpin loops protruding from the ankyrin repeats. Inspired by this structural insight, we replaced the hairpin loop with an unrelated short “SGGG” motif. Notably, substituting the loops between repeats 1/2 and 2/3 significantly enhanced *At*GORK activity, whereas similar replacement between repeats 4/5 and 5/6 impaired *At*GORK function (Fig. 3D and 3E). To rule out the possibility that these mutations affected channel trafficking, we also performed GFP fusion analysis on the resultant mutants (Fig. S6A and S6B), which confirmed normal surface expression levels. This aligns well with findings from ANK truncational studies (Fig. 3B and 3C), confirming that disrupting ANK dimeric interactions releases *At*GORK from autoinhibition and thus allowing channel activation. However, these observations also suggest that the ANK repeats in GORK have a more complex role than initially anticipated: they are not only essential for maintaining the autoinhibited state but also play a pivotal role in stabilizing the open state during channel activation.

### Mutational test on conserved acidic residues at the C-linker/TMD interface

**Figure 4.**
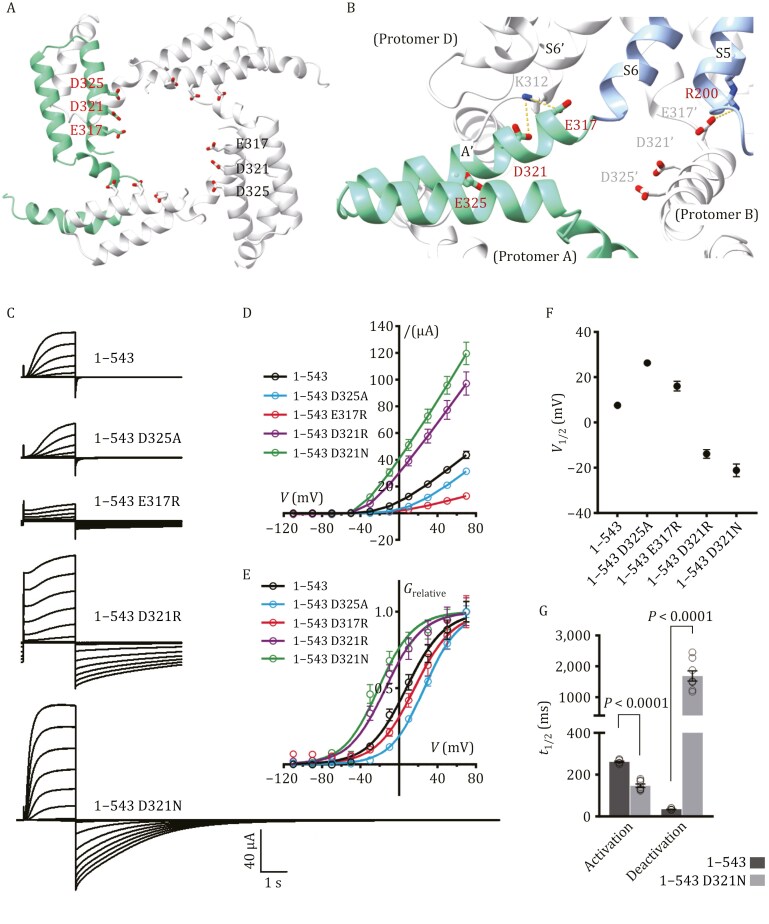
Mutational tests on conserved acidic residues at the C-linker/TMD interface. (A) Ribbons drawing of the C-linkers in tetrameric assembly, highlighting negatively charged residues (E317, D321, D325) in sticks. (B) Close-up view of the interface between the C-linker and TMD. E317 and D321 (C-linker of protomer A) form ionic interactions with K312 (Helix S6′ of protomer D), while R200 (Helix S5 of protomer A) forms a salt bridge with E317 from its adjacent protomer B. (C–G) Electrophysiological analyses of E317, D321, and D325 mutations in *At*GORK^543^. Representative current traces (C) and steady-state current–voltage (*I–V*) relations (D) are shown. Relative conductance–voltage (*G*_*relative*_–*V*) curves (E) and half-activation voltage (V_1/2_) values (F) were generated through Boltzmann sigmoidal fitting (outliers excluded). Conductance was calculated using the equation *G* = *I*/(*V* − *E*_*K*_), where *I* is the steady-state current, *V* is the test potential, and *E*_*K*_ (−58 mV) was derived from the Nernst equation based on intracellular (~100 mmol/L) and extracellular (10 mmol/L) K^+^ concentration in the oocyte TEVC recordings. Relative conductance was calculated by normalization to the maximal conductance. Half-time (t_1/2_) for activation at +70 mV and deactivation at −110 mV, calculated as ln (2)·τ where τ is the time constant from single-exponential decay fitting, is shown in (G). Data are mean ± SEM, *n* ≥ 8. Significance analysis was performed using unpaired Student’s *t*-test, with *P*-values displayed on the bar charts.

The C-linkers assemble into a disc-shaped gating ring via “elbow-on-shoulder” interactions (Flynn et al., 2007), directly contacting the TMD and playing a crucial role in channel gating. Conserved negatively charged residues, including E317, D321, and D325, are positioned on the C-linker A′ helix, forming a negatively charged ring at the entrance of the ion-conducting pathway (Fig. 4A) and engaging in an intricate interaction nexus with conserved K/R from the TMD (Fig. 4B). Specifically, E317 and D321 (C-linker of protomer A) form ionic interactions with K312 (Helix S6′ of protomer D), while R200 (Helix S5 of protomer A) forms a salt bridge with E317 from its adjacent protomer B. In such a fashion, the C-linker-mediated gating ring is connected with the VSD and PD within the TMD through their domain-swapped interfaces. These three residues correspond to conserved R, D, and A in the inward K^+^ channels KAT1 (Clark et al., 2020) and AKT1 (Lu et al., 2022), respectively.

To further validate their functional role, we introduced the mutations, E317R, D321 (N or R), and D325A into both full-length *At*GORK and the ANK-truncated *At*GORK^543^ construct. In full-length GORK, E317R and D325A enhanced current amplitude, whereas D321N and D321R produced an even larger increase (Fig. S7A and S7B). In contrast, in the activatable ANK-truncated *At*GORK^543^, E317R and D325A partially reduced the current amplitude, yet it remained higher than that of the wild-type full-length *At*GORK, while D321N and D321R still significantly enhanced the currents (Fig. 4C and 4D). In addition, these mutants exhibited distinct alterations of half-activation voltage (V_1/2_) in their *G*_*relative*_*–V* relationships: V_1/2_ of E317R negatively shifted in full-length *At*GORK but showed slightly positively shifted in *At*GORK^543^; V_1/2_ of D325A had no apparent shift in the full-length construct but positively shifted in GORK^543^; and V_1/2_ of D321N and D321R negatively shifted in both constructs (Figs. 4E, 4F, S7C and S7D; Supporting information).

Intriguingly, E317R and both D321 mutations (D321N and D321R) exhibited significant changes in deactivation kinetics. Specifically, when the membrane potential was held at −110 mV following the test voltage, the tail current decay was markedly delayed (Figs. 4C and S7A). For D321N, the deactivation time constant (t_1/2_) was prolonged in both the full-length GORK and the ANK-truncated *At*GORK^543^, increasing from 75 ms to 1,992 ms, and from 34 ms to 1,687 ms, respectively (Figs. 4G and S7E). Moreover, D321N accelerated the activation kinetics, with t_1/2_ decreasing from 462 ms to 286 ms in the full-length, and from 261 ms to 147 ms in the truncated form respectively (Figs. 4G and S7E). Notably, inward K⁺ tail currents were observed during the −110 mV holding phase, indicating that these mutations disrupted the original outward rectification, thereby allowing inward K⁺ flow.

### Disrupting ANK-dimerization relieves constraints and restores C4 symmetry

Our structural studies reveal that ANK dimerization in full-length *At*GORK is directly responsible for reducing its tetrameric symmetry from C4 to C2, maintaining an autoinhibited state. Under resting conditions, GORK shows minimal basal currents, whereas ANK-truncated mutants, including *At*GORK^623^ and *At*GORK^543^, show significantly increased currents, suggesting that ANK truncations relieve autoinhibition and enhances GORK activity. Structural reconstructions of *At*GORK^623^ and *At*GORK^510^ resolved only the TMD portion, with their cytoplasmic regions remaining unresolved due to their intrinsic flexibility (Fig. S2). While the absence of density in these regions limits direct visualization of cytoplasmic symmetry, it likely reflects increased structural mobility upon ANK truncation, supporting the conclusion that ANK removal disrupts autoinhibitory interactions and converts GORK into an activatable state.

**Figure 5.**
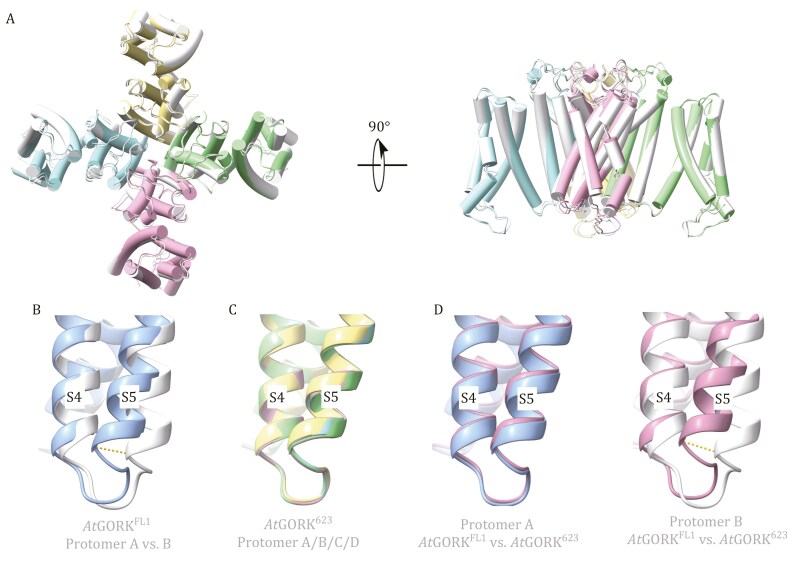
Structural comparison of the autoinhibited *At*GORK and the ANK-truncated variants. (A) Superimposition of the ANK-truncated variants (*At*GORK^623^ and *At*GORK^510^), with each protomer of *At*GORK^623^ shown in a different color, while all four protomers in *At*GORK^510^ are displayed in gray. Both truncated mutants showed nearly identical structures, with an RMSD of approximately 0.48 Å for their superimposed tetramers. (B–D) Close-up views of S4–S5 helix regions in the superimposed protomers of *At*GORK^FL1^ and *At*GORK^623^. (B) compares protomer A (blue) and protomer B (gray) of *At*GORK^FL1^, while (C) shows all four protomers of *At*GORK^623^. These comparisons reveal that *At*GORK^FL1^ exhibits quasi-C4 symmetry, whereas *At*GORK^623^ has strict C4 symmetry. The displacement of Cα position of Tyr196 in the C4–C5 region is approximately 3.8 Å (indicated by a yellow dashed line). (D) further compares protomers A and B of both *At*GORK^FL1^ and *At*GORK^623^, showing that the protomers in *At*GORK^623^ closely resemble protomer A in *At*GORK^FL1^, rather than protomer B. ANK truncation in *At*GORK^623^ restores C4 symmetry in the tetrameric assemblies.

Structural analysis also provided some clues regarding the restoration of C4 symmetry in the *At*GORK. Firstly, the structures of *At*GORK^623^ and *At*GORK^510^ are highly similar, with a root-mean-square deviation (RMSD) of approximately 0.48 Å for their superimposed tetramers (Fig. 5A). Importantly, the ANK-truncated protomers exhibit strict C4 symmetry, with RMSDs of approximately 0.16 Å/256 superimposed Cα. In contrast, the TMD of full-length *At*GORK^FL1^ displays quasi-C4 symmetry (essentially C2 symmetry), with subtle yet distinct differences between protomers A/C and B/D at the N-terminal end of helix S5, yielding an RMSD of approximately 0.75 Å/256 superimposed Cα (Fig. 5B and 5C). A comparison of protomers A and B in both *At*GORK^FL1^ and *At*GORK^623^ reveals that the protomers in *At*GORK^623^ closely resemble protomer A in *At*GORK^FL1^, rather than protomer B (Fig. 5D). This suggests that ANK dimerization results in C2 symmetry in the full-length *At*GORK^FL1^, whereas ANK truncation restores C4 symmetry in the *At*GORK^623^.

We also performed AlphaFold3-based modeling on *At*GORK^623^, showing that the TMD of the predicted *At*GORK^623^ structure is highly similar to that obtained by cryo-EM, with an RMSD of 1.97 Å for the superimposed tetramers. However, the subsequent portions, including the C-linker, CNBD, and ANK domains, are in a loose and relaxed conformation. Due to the lack of ANK dimerization, the overall structure exhibits a C4 symmetry, rather than a C2 symmetry (Fig. S8). Together with our structural and electrophysiological data, these observations support the role of ANK as a molecular switch for symmetry conversion in GORK, revealing a novel mechanism for channel regulation via symmetry conversions.

### A model for symmetry conversion in GORK regulation

**Figure 6.**
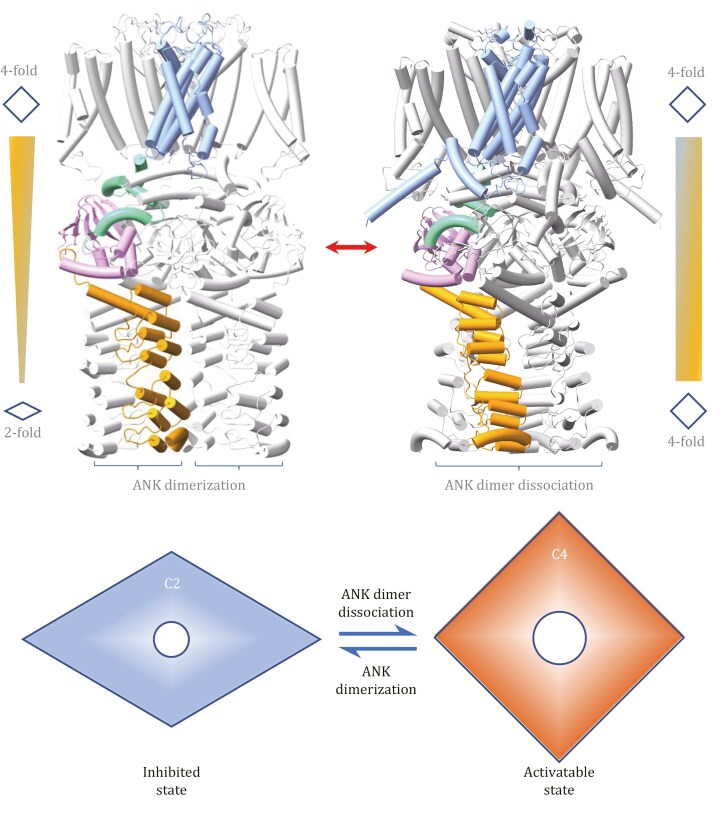
A model of symmetry conversion in plant GORK regulation. The GORK structure in the autoinhibited state (left, Cryo-EM) exhibits C2 symmetry, while in the activatable state (right, Alphafold3 prediction), it exhibits C4 symmetry. In brief, ANK dimerization reduces the symmetry of the GORK tetramer from C4 to C2, resulting in an autoinhibited state. Disrupting dimerization restores C4 symmetry, converting GORK to an activatable state. This dynamic symmetry conversion in the GORK tetramer provides a unique mechanism for regulating channel activity, enabling transitions between inhibited and activatable states—a distinctive feature of GORK family proteins containing the ANK domain. In such a way, ANK functions as a molecular switch, regulating the GORK channel in guard cells and controlling stomatal movement in response to environmental stimuli.

In summary, our analysis shows that full-length *At*GORK^FL^ adopts C2 symmetry due to ANK dimerization in the cytoplasm. Cryo-EM reconstruction of ANK-truncated *At*GORK^623^, together with AlphaFold3 modeling, indicates a restoration of C4 symmetry within the tetrameric assembly. This proposed C2-to-C4 symmetry conversion, potentially driven by the presence or absence of ANK-mediated dimerization, is hypothesized to regulate intermodular coupling and channel gating transitions. Accordingly, we propose that ANK dimerization holds the channel in an autoinhibited C2-symmetric state, whereas disrupting ANK interactions allows a transition to an activatable C4-symmetric configuration (Fig. 6).

However, we acknowledge that the cytoplasmic regions of the truncated constructs were not resolved in our cryo-EM maps, limiting direct structural validation of the restored symmetry. Thus, the proposed symmetry restoration remains a working model based on indirect structural and functional evidence, and awaits further confirmation through high-resolution structural studies of the cytoplasmic domains. Nevertheless, this model provides a plausible mechanistic framework for understanding how ANK functions as a molecular switch in GORK regulation.

## Discussion

Plant potassium channels are crucial for K^+^ transport and maintaining potassium homeostasis (Sharma et al., 2013). In *Arabidopsis*, nine Shaker-like potassium channels contain multiple modular domains in their architectures, including the pore-forming TMD, the regulatory C-linker, and CNBD, with some also containing ANK and KHA domains. Based on the presence of the ANK domain, these channels can be divided into two groups: ANK-free members (KAT1, KAT2, and KC1) and ANK-containing members (GORK, SKOR, AKT1, AKT2, AKT5, and AKT6). Currently, structural information are available for KAT1 (Clark et al., 2020; Li et al., 2020), SKOR (Li et al., 2023), and AKT1 (Dickinson et al., 2022; Lu et al., 2022), allowing us to conduct systematic comparative analysis

In ANK-free KAT1, structural studies show that it forms a symmetric tetramer with typical C4 symmetry throughout both the transmembrane and intracellular regions (Clark et al., 2020; Li et al., 2020), similar to animal cyclic nucleotide-gated channels (Xue et al., 2021). In contrast, GORK, which contains a unique ANK domain, exhibits a symmetry transition from C4 in the transmembrane region to C2 in the cytoplasmic region, resulting in distinct structural features. A similar symmetry reduction is observed in other ANK-containing potassium channels, such as AKT1 (Lu et al., 2022) and SKOR (Li et al., 2023). This reduction in symmetry is rare among ion channels, suggesting it may be a unique feature of ANK-containing channels linked to their regulatory mechanisms.

Our study provides the first clear-cut evidence that ANK acts as a molecular switch controlling GORK channel symmetry and activity. Structural analysis reveals that ANK dimerization holds GORK in an autoinhibited state at rest. ANK dimerization mediates interactions between protomers, reduces tetramer symmetry, and causes the displacement of internal, highly mobile modules, with neighboring CNBDH and C-linker shifting from C4 symmetry to C2. The C-linker adopts two different conformations: “kinked” and “flat” (Fig. 2B). The “kinked” conformation is common in C4 symmetric channels, while the “flat” conformation is unique to ANK-containing channels with reduced symmetry. Subtle yet distinct differences in the S5 helices between adjacent protomers in *At*GORK^FL1^ renders a quasi-C4 symmetry in the TMD (Fig. 5B). The reduction in GORK symmetry signifies an autoinhibited state, supported by the observed basal electrophysiological activity.

Our ANK truncation experiments showed that ANK removal converts GORK from an autoinhibited to an activatable state, as evidenced by enhanced activities in the truncated *At*GORK^543^ and *At*GORK^623^ variants upon membrane depolarization (Fig. 3B–E). Structural analysis further revealed that the TMD restores strict C4 symmetry, while the C-linker and CNBDH become highly mobile in these ANK-truncated mutants. Additionally, AlphaFold3-based modeling confirmed that disrupting ANK dimerization relieves constraints and restores C4 symmetry in the tetramer, enabling GORK transition to an activatable state. Thus, ANK serves as a molecular switch to regulate GORK activity by controlling the symmetry conversion of its tetrameric assembly.

Ankyrin domains also occur in TRPV channels, where they form a “petal-like” arrangement in the cytoplasm and act independently rather than directly participating in channel assembly (Kwon et al., 2023). Typically, these domains consist of six ankyrin repeats, forming a “palm-and-finger” structure, whose concave surface binds cytoplasmic proteins or ligands. Unlike GORK, TRPV gating depends on ANK conformational changes, such as rigid-body rotation. In TRPV4, RhoA binding to the ANK domain inhibits channel activity, whereas calmodulin binding enhances it, highlighting the versatile roles of ANKs in fine-tuning channel activity and coordinating intracellular signaling.

The autoinhibited‐to‐activatable transition of the GORK channel is governed by the dimerization state of its ANK domain, yet the molecular triggers of this dimerization and its reversal remain unknown. A recent study suggests that the cytosolic N-terminus of GORK functions as a “safety catch,” restricting channel activation by stabilizing the closed state (Zhang et al., 2025). In the truncated mutant GORK^Δ23^, removal of the N-terminal segment not only relieves autoinhibition and markedly enhances channel activity, but also restores C4 symmetry in the preopened conformation. This provides additional evidence for a C2–C4 symmetry switch during channel assembly. These findings suggest that both the N- and C-termini are crucial for GORK regulation, although the molecular mechanism of their coordination remains to be elucidated. The same study also performed ANK truncation (GORK^Δ527^), but observed no increase in channel activity (Zhang et al., 2025). We believe this discrepancy is mainly due to differences in the specific truncation sites, as the α-helix (spanning residues 520–543) connecting the ANK domain to the CNBDH—preserved in our constructs—is essential for stabilizing the activated state of GORK (Fig. 3B and 3C).

Previous studies showed that phosphorylation affects ANK-mediated protein–protein interactions and channel activity (Kwon et al., 2023; Li et al., 2006; Sedgwick and Smerdon, 1999). Several protein kinases, including CPK21 (van Kleeff et al., 2018) and CPK33 (Corratgé-Faillie et al., 2017), have been identified as candidates for regulating GORK activity, with phosphorylation sites, such as S649 in the ANK domain (Lefoulon et al., 2016). However, the molecular mechanisms of these kinases require further investigation. A parallel case is AKT1, which is activated by CIPK23 and CBL1, with multiple potential phosphorylation sites identified (including within its ANK domain). The low-resolution structure of AKT1 co-expressed with CIPK23 and CBL1 shows C4 symmetry in the CNBDs, though it remains unclear whether this is due to ANK phosphorylation (Lu et al., 2022).

Autoinhibition is an intrinsic mechanism that maintains ion channels and transporters in an inactive state, conserving energy in the resting state. In plants, different proteins achieve this through distinct molecular strategies. For example, SLAC1—a guard‐cell anion channel essential for stomatal closure—is held in its autoinhibited state by interactions between its N-terminal domain and the pore-forming transmembrane region; phosphorylation relieves this inhibition, rendering the channel activatable (Qin et al., 2024). By contrast, GORK utilizes its ANK domain as a molecular switch: ANK dimerization reduces the tetramer symmetry (from C4 to C2) to ensure autoinhibition, while disrupting ANK dimerization restores C4 symmetry and allows GORK becoming activatable. Together, these cases highlight the diversity of autoinhibition mechanisms and shed light on their roles in plant physiology.

In summary, our study reveals that ANK regulates GORK channel activity through tetrameric symmetry conversion. Under resting conditions, ANK dimerization maintains GORK in a C2 symmetric conformation, maintaining it in a preactivated, autoinhibited state. In response to environmental stimuli, GORK undergoes a symmetry conversion and runs into an activatable state, enabling K^+^ efflux and stomatal closure. This mechanism provides new molecular insights into stomatal regulation and offers potential avenues for developing improved crop varieties through ANK engineering.

## Supplementary data

Supplementary data is available at *Protein & Cell* online https://doi.org/10.1093/procel/pwaf067.

pwaf067_Supplementary_Data
