# Supplementary material for: Structural and mechanistic insights into symmetry conversion in plant GORK K+ channel regulation
Source: Protein Cell. 2025 Aug 3;16(12):1035–47. doi: 10.1093/procel/pwaf067 (PMC12742848; doi:10.1093/procel/pwaf067)
Supplement: pwaf067_Supplementary_Data [file pwaf067_supplementary_data.pdf]

Methods

Expression and purification of the GORK proteins

The genes of *Arabidopsis* full-length *At*GORK (AT5G37500) and truncated variants (*At*GORK^{623}, residues 1-623 and *At*GORK^{510}, residues 1-510) were cloned into a modified pEG BacMam vector, which included a PreScission protease cleavage site, GFP, an 8× His tag, twin-Strep tag, and Flag tag at the N-terminus. Recombinant proteins were expressed in HEK293F cells. In short, baculovirus was generated in *Sf9* insect cells, and P2 viruses were used to infect HEK293F cells. Cells were cultured in suspension, supplemented with 1% (v/v) fetal bovine serum, and maintained at 37 °C with 70% humidity and 6% CO_2. Cells were infected with P2 viruses at a density of approximately 2.6×10^6 cells/mL, and supplemented with 10 mM sodium butyrate upon 8 hours post-infection, and continued to grow at 30°C for another 50 hours. Cells were harvested, centrifuged, and stored at -80°C.

Harvested cells from 300 mL of culture were resuspended in extraction buffer (20 mM HEPES-Na pH 8.0, 150 mM KCl, 10 mM DDM, 2 mM cholesteryl hemisuccinate (CHS) supplemented with protease inhibitors (2 µg/mL pepstatin, 2 µg/mL aprotinin, 2 µg/mL leupeptin and 1 mM phenylmethylsulfonyl fluoride) and incubated at 4°C for 2 hours. Solubilized membranes were clarified by centrifugation at 41,000 rpm for 1 hour, and the supernatant was loaded onto a Streptactin Beads 4FF column. Following a 5-column volume buffer wash, proteins were eluted with solubilization buffer (20 mM HEPES-Na pH 8.0, 150 mM KCl, 0.02% GDN, and 2.5 mM desthiobiotin). After tag removal with TEV protease, the proteins were further purified using a Superose-6 column in solubilization buffer (20 mM HEPES-Na pH 8.0, 150 mM KCl, 0.006% GDN). The elution was analyzed by SDS-PAGE, and the peak fractions of the purified protein were pooled and concentrated to ~3.5 mg/mL.

For the preparation of truncated truncated versions (*At*GORK^{623} and *At*GORK^{510}), we followed similar procedures as above.

Cryo-EM grid preparation and data acquisition

The protein sample (4 µl, ~3.5 mg/ml) was applied to freshly glow-discharged holy carbon film grids (Quantifoil, Cu, R1.2/1.3, 300 mesh) and blotted for 6.5 seconds at 100% humidity and 4°C using a Vitrobot Mark IV (Thermo Fisher Scientific), followed by plunge freezing into liquid ethane.

All the films were collected on a Titan Krios transmission electron microscope (Thermo Fisher Scientific, USA) operated at 300 kV equipped with a Gatan K2 or K3 Summit direct detection camera (Gatan Company, USA). Movies were recorded at 22,500× (for K3, corresponding 1.06 Å per pixel) or 130,000 × (for K2 summit, corresponding 1.04 Å per pixel) nominal magnifications, using SerialEM software with a beam-image shift method (Mastronarde, 2005; Wu et al., 2019), at a total dose of 50 e-/Å2 distributed over 32 frames, with defocus values between -1.2 and -2.2 µm.

Data processing and model building

All image processing steps were conducted using either CryoSPARC 3.1 (Punjani et al., 2017) or RELION 3.0 (Zivanov et al., 2018). CryoSPARC 3.1 was primarily used for reconstruction, while RELION 3.0 was employed for alignment-free 3D classification to identify an alternate conformation (*At*GORK^{FL2}) of the wild-type structure. Angular information was converted using PyEM, and map analysis and adjustments were performed with UCSF Chimera (Pettersen et al., 2004).

Raw movie frames were aligned using a dose-weighted patch alignment method, followed by CTF estimation. Particle picking was performed an oval template (88 × 168 Å), and coordinates were screened using normalized cross-correlation and local power scores to eliminate false positives. Particles were then down-sampled to a quarter size and classified based on shape (circular for top-view projections and T-shaped for side-view projections) after initial 2D classification to improve classification efficacy. A preliminary 3D reconstruction was generated from a small subset of particles.

*At*GORK^{FL1} was constructed with Nu-refinement using C2 symmetry with 156,313 particles, while *At*GORK^{FL2} used 39,551 particles with C1 symmetry. The resolutions were 3.4 Å for *At*GORK^{FL1} and 4.3 Å for *At*GORK^{FL2}, respectively. The alphafold2-predicted *Arabidopsis* GORK structure was used as the initial model, fitted

by individual domains in UCSF ChimeraX (Pettersen et al., 2021), and refined iteratively in Coot (Emsley et al., 2010) and PHENIX (Liebschner et al., 2019), and corrected iteratively. For the truncated *At*GORK^{623} and *At*GORK^{510}, the TMD structure from *At*GORK^{FL1} was used as the initial model, followed by iterative refinement and iterative adjustments in Coot and PHENIX. The resolutions were 3.2 Å for *At*GORK^{623} and 3.4 Å for *At*GORK^{510}, respectively. All figures were generated using UCSF ChimeraX.

Electrophysiology

The electrophysiological experiments were performed as previously described (Deng et al., 2021). Briefly, untagged coding sequences of *At*GORK wild type or mutated variants were cloned into the pGHME2 vector for expression in *Xenopus* oocytes. cRNAs were synthesized using T7 polymerase with linearized plasmid DNA templates. Oocyte sacs were extracted and digested with 0.2 mg/ml collagenase (Sigma) in OR2 buffer (82.5 mM NaCl, 2.5 mM KCl, 1 mM $MgCl_2$, and 5 mM HEPES-Na pH 7.5). For expression, 36 ng of wild-type GORK cRNA was injected per oocyte, with mutant cRNA doses adjusted to equimolar concentrations. Injected oocytes were incubated at 18°C in ND96 buffer (96 mM NaCl, 1.8 mM $CaCl_2$, 1 mM $MgCl_2$, 2 mM KCl, and 5 mM HEPES-Na pH 7.5) for 48 hours before use.

TEVC recordings were performed using an OC-725C amplifier (Warner Instruments) and Digidata 1550B (Molecular Devices) controlled by pClamp software. The bath solution contained (in mM): 10 KCl, 90 NaCl, 1 $CaCl_2$, 2 $MgCl_2$, and 10 Tris/MES (pH 7.4), with osmolarity adjusted to ~220 mOsmol/kg using D-mannitol. Microelectrodes (0.5-1 MΩ resistance) were filled with 3 M KCl, and agar bridges served as bath electrodes. Voltage steps from -110 mV to +70 mV (20-mV increments, 2,000 ms duration) were applied, followed by -110 mV clamping for 450 ms, 4,000 ms, or 16,000 ms depending on deactivation kinetics. Steady-state currents were measured at 50 ms before the end of each step. Data were analyzed using Clampfit 10.6 (Molecular Devices), Prism 8.0 (GraphPad), and Origin 2021.

Fluorescence and confocal imaging

To assess the cell-surface expression of full-length and C-terminal truncated GORK channels in *Xenopus* oocytes, GFP was fused to the N-terminus of each construct. The corresponding cRNAs were injected into oocytes, which were then incubated at 18°C for ~48 hours in ND96 buffer (detailed in the *Electrophysiology* section). GFP-tagged protein expression was verified using a confocal laser scanning microscope (Zeiss LSM 980), and fluorescence intensities were quantified with ImageJ. To minimize variability in expression levels due to experimental factors (e.g., cRNA injection dose or incubation time), all experiments were performed in parallel.

Data availability: All data generated or analysed in this paper are presented in the main text, figures and the extended data figures and supplementary videos, or are available from the corresponding author upon request. The cryo-EM maps of the *At*GORK full-length (*At*GORK^{FL1} and *At*GORK^{FL2}) and truncated version (*At*GORK^{623} and *At*GORK^{510}) have been deposited in the Electron Microscopy Data Bank with accession codes EMD-62338, EMD-37500, EMD-62337 and EMD-62339, respectively, and their structural models have been deposited in the PDB with accession codes 9KHF, 8WFZ, 9KHE and 9KHG, respectively (Supplementary Table S1).

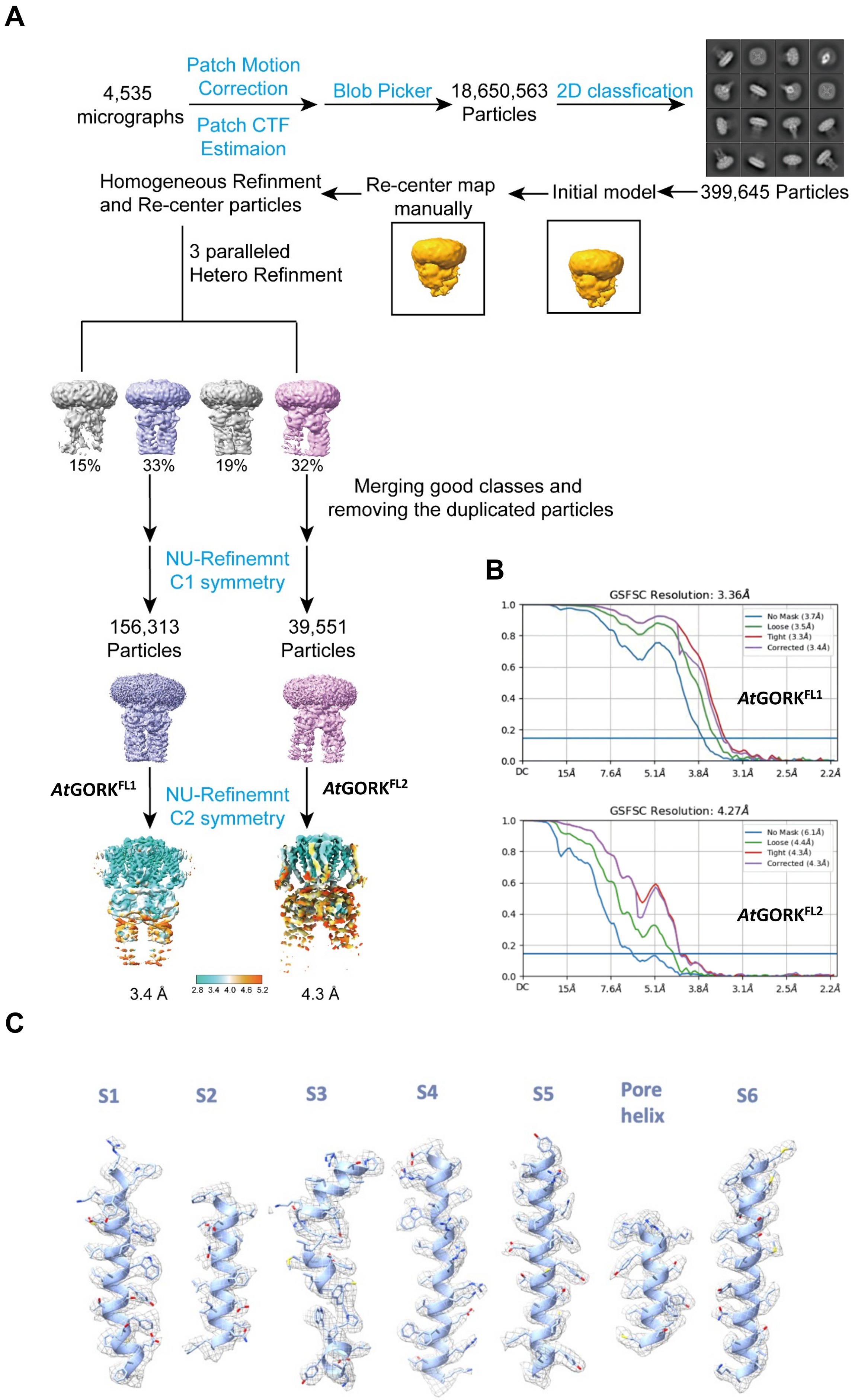
A
4,535
micrographs
Patch Motion
Correction
Patch CTF
Estimaion
Blob Picker
18,650,563
Particles
2D classfication
399,645 Particles
Initial model
Re-center map
manually
Homogeneous Refinment
and Re-center particles
3 paralleled
Hetero Refinment
15%
33%
19%
32%
Merging good classes and
removing the duplicated particles
NU-Refinemnt
C1 symmetry
156,313
Particles
39,551
Particles
AtGORKFL1
AtGORKFL2
NU-Refinemnt
C2 symmetry
3.4 Å
2.8 3.4 4.0 4.6 5.2
4.3 Å
B
GSFSC Resolution: 3.36Å
No Mask (3.7Å)
Loose (3.5Å)
Tight (3.3Å)
Corrected (3.4Å)
AtGORKFL1
GSFSC Resolution: 4.27Å
No Mask (6.1Å)
Loose (4.4Å)
Tight (4.3Å)
Corrected (4.3Å)
AtGORKFL2
C
S1
S2
S3
S4
S5
Pore
helix
S6

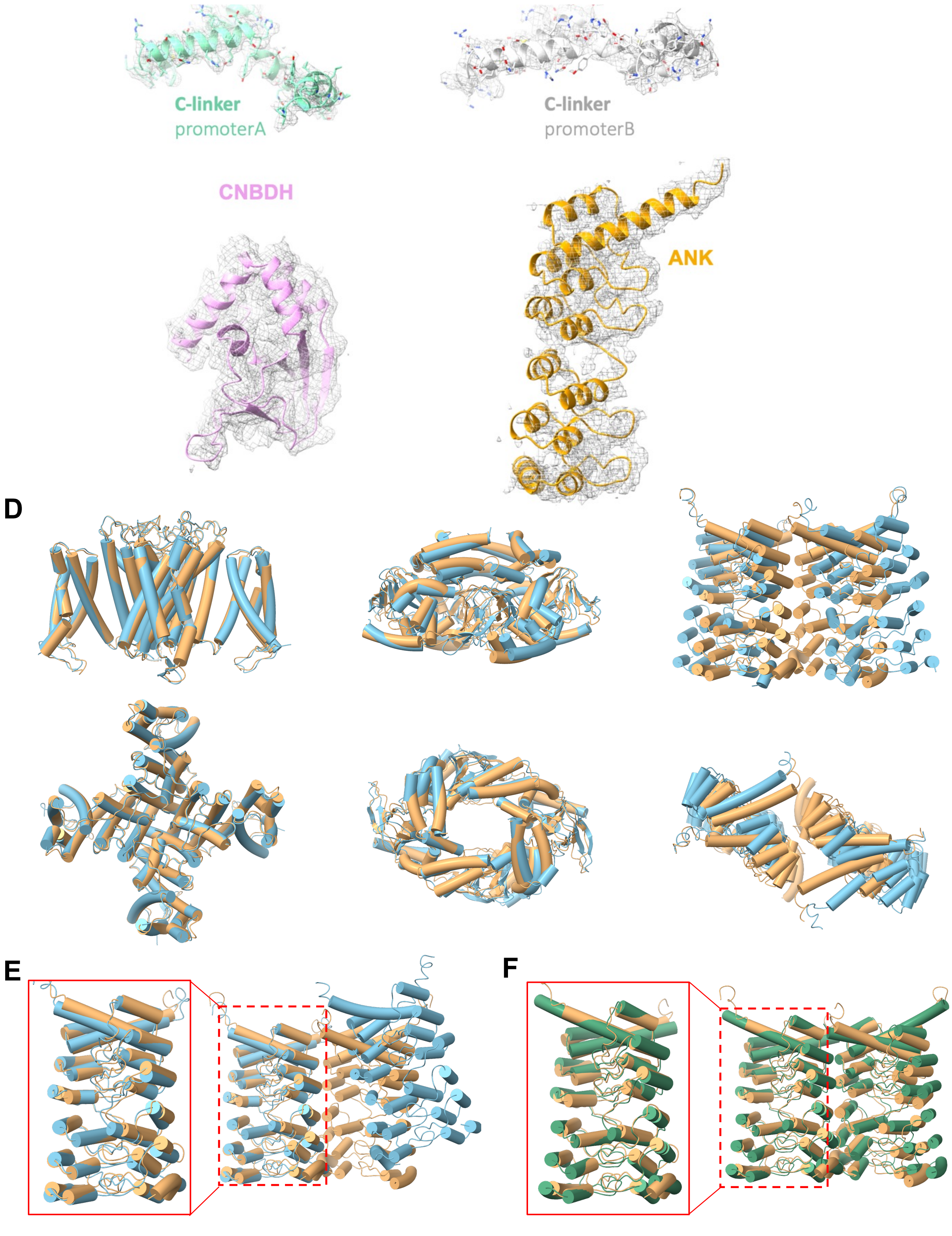

Figure S1. Structural determination of full-length *Arabidopsis* GORK (*At*GORK^{FL}).

(**A**) Workflow for image processing of full-length *Arabidopsis* GORK. (**B**) Fourier shell correlation (FSC) curve indicating overall resolution of 3.4 Å for *At*GORK^{FL1} and 4.3 Å for *At*GORK^{FL2}, as estimated using the 0.143 cut-off criterion (dotted line). (**C**) Representative cryo-EM density map showing TM segments (S1-S6) and domains (C-linker, CNBDH and ANK). (**D**) Structural comparison of domains in *At*GORK^{FL1} (light orange) and *At*GORK^{FL2} (light blue). (**E**) Superimposition of ANK dimers in *At*GORK^{FL1} (light orange) and *At*GORK^{FL2} (light blue), with a RMSD of 1.21 Å/dimer. (**F**) Superimposition of ANK dimers in *At*GORK^{FL1} (light orange) and *At*SKORK (purple, 8jet), with a RMSD of 1.33 Å/dimer. Despite variations in ANK conformation across the tetramer, the dimeric interaction is conserved, indicating that ANK dimerization is a fundamental structural feature of potassium channels with ANK domains.

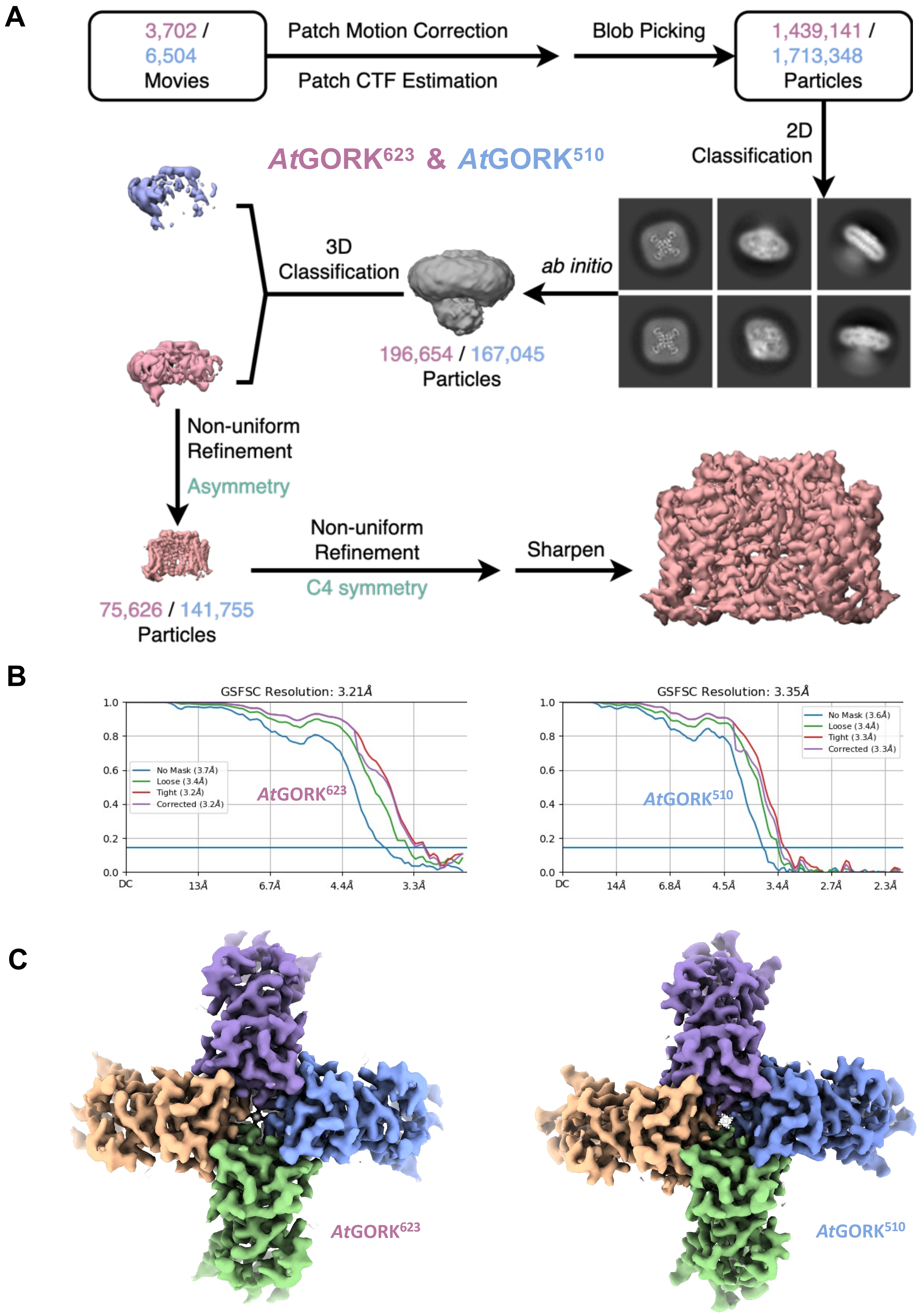

Figure S2. Structural determination of the truncated *At*GORK^{623} and *At*GORK^{510}.

(**A**) Workflow for image processing of the truncated *At*GORK^{623} (residues 1-623) and *At*GORK^{510} (residues 1-510). (**B**) Fourier shell correlation (FSC) curve indicating overall resolution of 3.2 Å for *At*GORK^{623} and 3.4 Å for *At*GORK^{510}, as estimated using the 0.143 cut-off criterion (dotted line). (**C**) Cryo-EM structures of *At*GORK^{623} (3.2 Å, left) and *At*GORK^{510} (3.4 Å, right).

```
                                                                                                                                      S1
Arabidopsis_GORK      --------------MGRL-----RR---R-------QEI-------IDHEEEESNDDVSSRRGKLSLAETFRWLDSSEHRRIET--------DGH--NDYKYIIHPK-NRWYKAWEMFIL   73
Brassica_GORK         --------------MGCL-----RR---R-------QES-------IAE-EDDINDDVSRRRGRFSLAESFRWLDSPEHLKDDS--------DGH--NEYPWIIKPS-IRWYKAWELFIL   72
Zingiber_GORK         MERDSSRRK-WKRTMSAVMAPRPSEAEEEAVGLASDGGSGGGDEYTVEEVHDLIG---SSRGSRQSVLADESRR------------------ASGDELLLRFVFRPD-NGWYETWTQFIM   97
Elaeis_GORK           -------------------------------MSPRAKNGSPEIEYEL---EEPIA---SSRGIRLFLLTSEFALGPLRRRRAT----------SQEKLLERFVIEPD-NRWYQLWTRFIL   72
Phoenix_GORK          -------------------------------MSPHAKNESQEIEYEL---EEPMG---SSRGIRWSLITSDFAIGPLRRR-AH----------SHEKLLERFVIEPD-NKWYQLWTRFIL   71
Oryza_GORK            MGRGIGSKRRVEDDDGENMPGRK---KKE--EEEEEEDDDGEEEYEVDVVRDRIG---SSRGSRLALFGSDLRLGRFRPRRRRV--APV---DGDDGIFQDFVIDPD-NKWYRLWTRFIL   106
Brachypodium_GORK     MGRGLGSKSRVES-------------------------EVEEEEYEVEVVPDRLK---SSRNSRLALFGSELRLDRFRPRRRRRRRAAA---DGEDGFFHDLIIHPE-NKWYRIWSRFIL   88
Panicum_GORK          MGRGLGSKRRVAEGDTK----------------PEEEEEGSSEEYEVEVVRDHIA---SSRGSRLALFGSDLRLGRFRPRRRRA---------AAEGFFQDLVIHPD-NRWYRLWTKFIL   91
Papaver_GORK          MPD----------HLHHL-----HRHHHERERDKREEDEDETEEFKVEKLKDRIL---SSRGSRFDLITNELGLDNQ-TRRKISREDFI---NGIR----GFVIHPD-NRWYLIWTRIIL   93
Amborella_GORK        -------------------------------MCPGSEERDMQEEYQVEEFRERIN---SSRGSRFALLSELTKR---------GGGSQERLIQGFRGCTQSVIIHPD-SRWYQVWTFIIL   76
Vulgaris_GORK         ---------MTPKQLGRIISGKTMSRIREEGGSTSSSSSNRIEDFVVEKPRERLT---SSRGSRLDLLVNELNDES--TRRQFSREGFF---LGVKDLYHGLVIHPEDNRWYKAWDKFIL   103
Chenopodium_GORK      ---------MKPKQIARIMMTRG--REERMEGSTSSSSSNREEDFMVERPRERLY---ASVGSRFNLLVTEFSHES--SQRQFTREGFF---VGVKDLYHGLVIHPEHNRWYKAWDNIIL   101
Lactuca_GORK          ------------MRIGST---------ADERRRKAVEDEVVGEEYVVEDVRDHDHGRLTKRRSRFTLLAGDLDLDP--SRRDFNTEK---FIDGIRGCSPALFIHPQ-NSWYKAWEKFIL   93
Daucus_GORK           ----------------MSNRRRMEEEKRRRRKGQDSEDSSNDEEYEVEDVREDVR---ESRGHHMARIASEFEAATEAYRRIYSREHIF---KGLTDLSRGCSIHPD-NRWYHAWEKFIL   97
Medicago_GORK         ---M---KMKEEEDEGEKNRINNQTATCSGSSSLTPSSSSDEREYQVQDLRDKLK---SSRGSRFNLIEKELGLKI--GWRKFSR----------RTLLHEFVINPL-NRWYRAWLKFIL   98
Prosopis_GORK         MPGN---RLTEEAVTEEREEKRKQMLSTSRSSSSSSSSSSDDREYELEDLRDRLK---SSRGSRFQLIENELKE-L--NQKKFSRHV----IDGIRGLSKDFIIHPE-NRWYRAWTKFIL   106
Nicotiana_GORK        -------------------------MTRVAEEVLETMNMNGGVEYEVEDVRDKIE---SSRGSRFQLIEDDL-GLNL-FRRRISRL--------KINLSQVFIIHPE-NRWYRMWENFIL   81
Solanum_GORK          --------------------------MTRGEEVLEIDINGGRAEYEIEDMRDKME---SSRGSRFKLIENDLVGADM-FSRRRRKFSRESLLNGLKDLSQGFVIHPE-NRWYRMWENFIL   89
Coffea_GORK           -------------------------MGRGNSHREEGESRNGEGEYEVEDLRDRIK---SSRGSRFELIEHELGL-DS-THRRFSRQSVI---NGLKDLSQGLVIHPD-NRWYRAWEKFIL   86
Sesamum_GORK          --MG---SFKKSSF--RN-----TDESSANGEESSLNGVNGGEEYMVEDLRERIK---SSRGSRFTLIENELGLIDS-ARRRVSRQTVI---DGLKDLSQGLFIHPD-NRWYRAWEKFIL   100
Cucumis_GORK          -----------MAVSG-------GREKEEEAGTHRRSDSDGEVEYRVESVRERIS---SSRGSRFNLIQRQLRID--QSRRRFSRENLI---NGIK----CLVILPD-SRWYRAWTKFIL   89
Vitis_GORK            --MN---RFMY--LVGRG----------------QVVDDEDDGDFQMPDLRERIK---GSSASVLNMFNGDLGLR------RFSGGSCF---NGIK----GFIILPD-NWWYRTWTKFIL   80
Cannabis_GORK         ---M---SFAAANGIGRV-----DQNDERDHDMVSSSSDGDEEEFELQDIRDRIK---SSRGSRFNLIANEFGLASAI--KKFSRENVM---NGIRDLSNGVVIHPD-NRWYRAWTKFIL   100
Gossypium_GORK        -----------MGDIGRS-----RRV-----RDERCATMANEEEYEVEDFREGNK---SSRGSRFNLIANELGLVAARARMNLSRQSVL---NGIKDLSDGP-IHPD-NRWYRAWTKFIL   91
                                                                    :    :                                                   : *    **  *  :*:

                              S1                        S2                            S3a        S3b      S3c                 S4
Arabidopsis_GORK      VWAIYSSLFTPMEFGFFRGLPE--RLFVLDIVGQIAFLVDIVLQFFVAYRDTQTYRTVYKPTRIAFRYLKSHFLMDFIGCFPWDLIYKASGKHELVRYLLWIRLFRVRKVVEFFQRLEKD   191
Brassica_GORK         VWAIYSSLFTPMEFGFFRGLPE--NLFILDIVGQIAFLVDIVLQFFVAFQDKHTYRIDSKPTHIALRYLKSHFFLDLVSCFPWDLIYKASGKHEVVRYILWIRLFRVRKVIEFFQRLEKD   190
Zingiber_GORK         LWAVYSSFFTPLEFGFFRGLPK--NLIFLDMTGQVAFLVDIIVNFFLAYRDSLTYRMIYSPTSIAFRYLKSSFVFDFLACVPWDYIYKLSGRKEEVRYLLWIRLIRVRKVTDFFQRMEKD   215
Elaeis_GORK           VWALYSSFFTPIEFGFFRGLPN--NLFWLDFAGQVAFLVDIFVQFLVAYRDSHTYRMIYEPTSIAVRYAKSSFVFDLLGCFPWDAIYRACGRKEEVRYLLWIRLTRVRKVTDFFQKMEKD   190
Phoenix_GORK          VWALYSSFFTPIEFGFFRGLPN--NLFWLDFAGQVAFLVDIFVQFLVAYRDSHTYRMIYKPTSIAVRYVKSSFVFDLLGCFPWDVIYKACGNKEEVRYLLWIRLTRVRKVTDFFQKMEKD   189
Oryza_GORK            VWAVYSSFFTPLEFGFFRGLPR--NLFFLDIAGQIAFLIDIVLRFFVAYRDPDTYRMVHNPTSIALRYCKSSFIFDLLGCFPWDAIYKACGSKEEVRYLLWIRLTRAMKVTEFFRSMEKD   224
Brachypodium_GORK     VWAVYSSFFTPFEFGFFRGLPK--RLFFLDIAGQIAFLIDIVLKFFVAYRDPDTYRMVYNPTSIALRYCKSSFIFDLLGCFPWDVIYKACGSREEVRSLLWIRLTRALKVTEFFKDLEKD   206
Panicum_GORK          VWAVYSSFFTPLEFAFFRGLPR--KLFLLDIAGQIAFLIDIVVKFFVAYRDPDTYRIVYDPTAIALRYFKSSFIFDLLGCFPWDVIYKACGRKEEVRYLLWIRLTRALKVTEFFWHLEKD   209
Papaver_GORK          IWAIYSSFFTPVEFGFFRGLPD--KLFILDIAGQFAFFVDIIIQFFVAFRDPQTYRMVSKRTTIALRYLKSSFILDLLACCPWDLIYASCGKKEEVRYLLWIRLYRVRKVIEFFQHMEKD   211
Amborella_GORK        MWAIYSSFFTPLEFGFFRGLPK--NLVFLEVAGQIAFLVDILFNFFLAYRDSQTYRMVYKRSDIALRYAKSCFLVDLFGCLPWDAIYKASGRKEVVRYLLWVRLSRLQKVTNFFQRLEKD   194
Vulgaris_GORK         LWAIYSSFFTPMEFGFFRGLPE--NLFVLDIVGQVAFLLDIVLQFFVGYRDKQTYRMVYQRPAIAFRYLKSTFVIDLLACMPWDLIYKASHHKEAVRYLLWIRLCRVRKVHYFLQKMEKD   221
Chenopodium_GORK      LWAIYSSFFTPMEFGFFRGLPE--NLFVLDIVGQVAFLMDIVLQFFVGYRDKQTYRMVYQRPAIAFRYLKSTFVLDLLACLPWDLIYKASHRKEAVRYMLWIRLCRVRKIHFFLQKLEKD   219
Lactuca_GORK          IWAVYSSFFTPMEFGFFRGLPK--NLYFLDIAGQIAFLIDVVLHFFIAYRDTQTYKMISNRNLIAVRYLKSHFLFDILACMPWDNIYRASGRKEEVRYLLLLRLVRVRKVLEFFSRLEKD   211
Daucus_GORK           LWAMYSSFFTPFEFGFFRGLPRNRNLFLLDIAGQTAFVIDIVLQFFVAYRDRQTYKMIYKRYPIAMRYLKSHFIIDFLGCLPWDIIYGATGNKEAVRCLLWIRLSRARKVLAFFQKLEKD   217
Medicago_GORK         LWAVYSSFFTPMEFGFFRGLPE--NLFILDIVGQIAFLVDIVLQFFVAYRDSQTYRMVYKRTPIALRYLKSTFVIDLLGCMPWDLIYKACGRREEVRYLLWIRLYRAERVVQFFRNLEKD   216
Prosopis_GORK         LWAIYSSFFTPMEFGFFRGLDE--DLFILDIIGQVAFLVDIIVQFFLAYRDSQTYRMIYKRAPIALRYLKSSFIFDLLGCMPWDIIYKASGRREGVRYLLWIRLYRVRKVTEFFQRLEKD   224
Nicotiana_GORK        IWSIYSSFFTPMEFAFFNGLPR--KLFLLDICGQIVFLVDIVLQFFVAYRDSQTYKMVYKRTPIALRYLKSHFILDFLSCMPWDIIYKAVGSKEEVRYLLWIRLSRARRITYFFQKMEKD   199
Solanum_GORK          IWSIYSSFFTPMEFAFFNGLPR--KLFLLDICGQIVFLVDIVIQFSVAYRDSQTYKMVYKRTPIALRYLKSHFIMDFLGCMPWDIIYKAVGSKEEVRYLLWIRLSRARRITYFFQKMEKD   207
Coffea_GORK           IWAVYSSFFTPFEFGFFRGLPR--KLLFMDIAGQAAFLVDIILQFLVAYRDSQTYRMVYKRTPIALRYIKSHFIIDLLGCMPWDFIYKAVGHKEVVRYFLWIRLSRVRKGNKFFQHMEKD   204
Sesamum_GORK          IWAIYSSFFTPMEFGFFRGLPK--NLFFLDIAGQVAFLLDIILQFFVTYRDSHSYKMVYNRNLIALRYLKSHFLPDLLGCMPWDIIYKAVGEKEEVRYLLWIRLTRVRKVTAFFQRMEKD   218
Cucumis_GORK          IWAVYSSFFTPMEFGFFRGLPE--NLFILDIVGQIAFLFDIVFQFFLAYRDKQTYRMVYKRSPIALKYLKSTFVTDLLSCMPWDILYKACGRREEVRYLLWIRLFRVRKVDAFFKTMEKD   207
Vitis_GORK            LWAVYSSFFTPMEFGFFRGLPE--DLVFLDIAGQIAFLIDIVLRFFLAYRDAHTYRMVYKRTSIALRYMKSSFVIDLICCLPWDIIYKACGRKEEVRYLLWIRLIRVCKVTDFFQNLEKD   198
Cannabis_GORK         LWAVYSSFFTPMEFGFFRGLNE--DLFVLDIVGQIAFLVDIVVLFFVSYRDSHTYRMVYKRTPIALRYLKSSFVIDLLCCLPWDIIYKKSGRHEAVRYLLWIRLSRVRKVTDFFHKLEKD   218
Gossypium_GORK        IWALYSSFFTPFEFGFFRGLPE--NLFVLDIAGQIAFLLDIILHFFLAYRDPQTYRMVYKRTSIAIRYLKSSFLIDLLGCMPWDIIYKASGRKEEVRYLLWIRLYRVHKVTAFFRKMEKD   209
                      :*::***:***.**.**.**     *  ::. ** .*..*:.. * : ::*  :*:   .   **.:* ** *. *:. * *** :*     :* ** :* :** *  :   *:  :***
```

```
                         S5                                    Pore-Helix                      S6
Arabidopsis_GORK    TRINYLFTRILKLLFVEVYCTHTAACIFYYLATTLPPENEGYTWIGSLKLGDYSYENFREIDLWKRYTTALYFAIVTMATVGYGDIHAVNLREMIFVMIYVSFDMVLGAYLIGNITALIV   311
Brassica_GORK       TRINYLFTRILKLIFVEVYCTHTAACIFYYLATTLPAENEGYTWIGSLKLGDYSYENFRKIDIWKRYTTSLYFAIVTMATVGYGDIHAVNLREMIFVMIYVSFDMVLGAYLIGNITALIV   310
Zingiber_GORK       IRINYLFTRIVKLIVVELYCTHTAACIFYYLATTLPASMEEYTWIGSLKLGDYSYSHFREIDLWRRYITSLYFAIVTMATVGYGDIHAVNTREMVFIMIYVSFDMILGAYLIGNMTALIV   335
Elaeis_GORK         IRINYLFTRIVKLIVVELYCTHTAACIFYYLATTLPASMEGYTWIGSLKLGDYSYSHFRDMDIAKRYITSLYFAIVTMATVGYGDIHAVNPREMVFIMIYVSFDMILGAYLIGNMTALIV   310
Phoenix_GORK        IRINYLFTRIVKLIVVELYCTHTAACIFYYLATTLPASMEGYTWMGSLKLGDYSYSHFRDIDLGKRYITSLYFAIVTMSTVGYGDIHAVNPREMVFIMIYVSFDMILGAYLIGNMTALIV   309
Oryza_GORK          IRINYLFTRIVKLIVVELYCTHTAACIFYYLATTLPESMEGYTWIGSLQLGDYSYSHFREIDLTKRYMTSLYFAIVTMATVGYGDIHAVNVREMIFIMIYVSFDMILGAYLIGNMTALIV   344
Brachypodium_GORK   IRVNYLFTRIVKLIVVELYCTHTAACIFYYLATTLPESMEGYTWIGSLKLGDYSYSNFREIDLAKRYMTSLYFAIVTMATVGYGDIHAVNVREMIFIMIYVSFDMILGAYLIGNMTALIV   326
Panicum_GORK        IRVNYLFTRIVKLIVVELYCTHTAACIFYYLATTLPESMEGYTWIGSLKLGDYSYANFREIDLAKRYITSLYFAIVTMATVGYGDIHAVNIREMIFVMIYVSFDMILGAYLIGNMTALIV   329
Papaver_GORK        IRINYLFTRIVKLLAVELYCTHTAACIFYYLATTLPAEKEGYTWIGSLKLGDYSYSNFREIDLGKRYIVSLYFAIVTMATVGYGEIHAVNVREMIFVMIYVSFDMILGAYLIGNMTALIV   331
Amborella_GORK      IRINYLFTRIVKLIVVELYCTHTAACIFYYLATTVPESEEGYTWIGSLTMGDYSYSHFREIDFIKRYLTSLYFAIVTMATVGYGDIHAVNPREMIFIMIFVSFDMILGAYLIGNMTALIV   314
Vulgaris_GORK       IRINYLFTRIVKLLTVELYCTHTAACIFYYLATTLPEREEGYTWIGSLTLGDYSYSHFREIDIWRRYTVSLYFAIVTMATVGYGDIHAVNMREMIFIMIYVSFDMVLGAYLIGNMTALIV   341
Chenopodium_GORK    IRINYLFTRIVKLLTVELYCTHTAACIFYYLATTIPEREEGYTWIGSLTLGDYSYSHFREIDLWRRYTTSLYFAIVTMATVGYGDIHAVNMREMIFIMIYVSFDMILGAYLIGNMTALIV   339
Lactuca_GORK        IRVNYLFSRIIKLIAVELYCTHTAACIFYYLATTLPAVEEGYTWIGSLKLGDYSYSNFREIDLWKRYTTSLYFAIVTMATVGYGEIHAVNMREMIFVMIYVSFDMVLGAYLIGNMTALIV   331
Daucus_GORK         IRIKYLFCRIVKLIVVEIYCTHTAACIFYYLATTLPAEKEGYTWIGSLKLGDYSYSNFREIDLWTRYITSLYFAIVTMVTVGYGDIHAVNLREMVFIMIFVSFDMVIGAYLIGNMTALIV   337
Medicago_GORK       IRVNYIIARIVKLLVVELYCTHTAACIFYYLATTLPESQEGYTWIGSLKLGDYAYSKFREIDIWKRYTTSLYFAIVTMATVGYGDIHAVNLREMIFIMIYVSFDMVLGAYLIGNMTALIV   336
Prosopis_GORK       IRINYMFTRIVKLIVVELYCTHSAACIFYYLATTLPPSQEGYTWIGSLQLGGYSYMNFREIDLWKRYTTSLYFAIVTMATVGYGDIHAVNMREMIFIMIYVSFDMVLGAYLIGNMTALIV   344
Nicotiana_GORK      IRINYLFTRIVKLITVELYCTHTAACIFYYLATTLSEQQEGYTWIGSLKLGDYSYSNFRDIDLWTRYTTSMYFAIVTMATVGYGDIHAVNLREMIFVMIYVSFDMILSAYLIGNMTALIV   319
Solanum_GORK        IRINYLFTRIVKLITVELYCTHTAACIFYFLATTLSEQQEGYTWIGSLKLGDYSYSNFRDIDLWTRYTTSMYFAIVTMATVGYGDIHAVNLREMIFVMIYVSFDMILSAYLIGNMTALIV   327
Coffea_GORK         IRINYLFTRIVKLIAVELYCTHTAACIFYYLATTLPPEKEGYTWIGSLKLGDYQYSSFRDIDLWKRYTTSMYFAIVTMATVGYGDIHAVNVREMIFIMIYVSFDMILGAYLIGNMTALIV   324
Sesamum_GORK        IRINYLFTRIVKLIAVELYCTHTAACIFYYLATTLPEEKEGYTWIGSLKLGDYSYAHFREIDIWKRYTTSMYFAIVTMATVGYGDIHAVNLREMIFIMIYVSFDMILGAYLIGNMTALIV   338
Cucumis_GORK        IRINYMFTRIVKLLVVELYCTHTAACIFYYLATTLPASEEGYTWIGSLKLGDYSYSHFREIDLWKRYTTSLYFAIVTMATVGYGDVHAVNLREMIFIMIYVSFDMVLGAYLIGNMTALIV   327
Vitis_GORK          TRINYMFTRILKLIAVELYCTHTAACVFYYLATTLPQSEEGYTWIGSLKLGDYSYSHFREIDIWKRYTTSLYFAIITMATVGYGDIHAVNLREMIFVMIYVSFDMILGAYLIGNMTALIV   318
Cannabis_GORK       IRINYIVTRIIKLIAVELYCTHTAACIFYYLATTLPPSKEGYTWIGSLKLGDYSYSSFRDIDLWKRYITSLYFAIVTMATVGYGDIHAVNMREMIFIMIYVSFDMILGAYLIGNMTALIV   338
Gossypium_GORK      IRINYLFTRIIKLIFVELYCTHTAACIFYYLATTLPREKEGYTWIGSLKLGDYSYSSFRDIDLWKRYTTSMYFAIVTMATVGYGDIHAVNMREMIFIMIYVSFDMVLGAYLIGNMTALIV   329
                     *::*:. **:**: **:****:***:**:****:    * ***:*** :*.* *  **.:*:  ** .::****:** *****::**** ***:*:**:*****::.******:*****

                     C-Linker/A'       C-linker/B'  C-linker/C'  C-linker/D'         Helix A   β1    β2     β3
Arabidopsis_GORK    KGSNTERFRDKMNDLISFMNRKKLGRDLRSQITGHVRLQYDSHYTDTVMLQDIPASIRAKIAQLLYLPYIKKVPLFKGCSTEFINQIVIRLHEEYFLPGEVITEQGNVVDHLYFVCEGLL   431
Brassica_GORK       KGSNTERFRDKMNDLASFMNRKKLRGDIRSQITHHVRLQYDSKFTNTVMLQDIPASIRAKIAQLLYTPYIEKIPLFKGCSSEFINQIVVRLHEEYFFPGEVITEQGNVVDHLYFVCEGSL   430
Zingiber_GORK       KGSKTERFRDKMKDLLNYMNRNKLGKEIRDQIKGHVRLQYESNYTEASILQDIPISIRAKISQTLYRPYIERVPLFKGCSTEFVNQIVIRLQEEFFLPGEVLLEQGNAVDQLYFVCYGVL   455
Elaeis_GORK         KGSKTERFRDRMKELIKYMNRNKLGKDIRDQIKGHVRLQYESSYTEASVLQDIPVSIRAKISQTLYKPYIENVPLFKGCSAEFIHQIIIKLQEEFFLPGEVILEQGNAVDQLYFVCHGKL   430
Phoenix_GORK        KGSKTERFRDRMKDLVKYMNRNKLGKEIRDQIKSHVRLQYESSYTEASVLQDLPVSIRAKISETLYKPYIDNVPLFSGCSEEFIHQIIIKLQEEFFLPGEVLLEQGDAVDQLYFVCHGKL   429
Oryza_GORK          KGSRTERFRDKMKEVIRYMNRNKLGKDIREQIKGHLRLQYESSYTEASVLQDIPVSIRAKISQTLYKPYIESIPLFKGCSAEFIQQIVIRLQEEFFLPGEVILEQGSAVDQLYFVCHGAL   464
Brachypodium_GORK   KGSRTERFRDKMKEVIRYMNRNKLGKEIREQIKGHLRLQYESSYTEASVLQDIPISIRAKISQTLYKPYIESTPLFKGCSAEFIQQIVIRLQEEFFLPGEVILEQGSAVDQIYFVCHGEL   446
Panicum_GORK        KGSRTERFRDKMKEVIRYMNRNKLGKEIREQIKGHLRLQYESSYTEASVLQDIPISIRAKISQTLYKPYVESVPLFKGCSAEFIQQIVIRLQEEFFLPGEVILEQGSAVDQLYFVCYGAL   449
Papaver_GORK        KGSKTERFRDKMADIMKYMNRNKLGKEIRDQIKGHLRLQYESSYTEAVVLQDIPISIRAKISQTLYRDFIEDVPLFKGCSPELKNQIVIRLHEEFFLPGEVIMEQGNIVDQIYFVCDGVL   451
Amborella_GORK      KGSKTERFRDKMTDLIKYMNRNKLEKEIRDQIKGHVRLQYESSYTEASVLQDIPVSIRAEISQSLYKSTIEAVPLLKGCSSEFINQIVIRLHEEFFLPGEVILEQGNAVDQIYFISHGVV   434
Vulgaris_GORK       KGSKTERFRDKMTEVMKYMNRNRLSRELRSQIKGHLRLQYESSYTEASILKDIPISLRAKISQTLYTPYIGRGPLFKGCSSEFINQIVTRVHEEFFLPGEVILEQGNVVDQLYFVCHGLL   461
Chenopodium_GORK    KGSKTERFRDKMTEVMKYMNRNRLSRDLRNQIKGHLRLQYESSYTEASVLQDIPISLRAKISQSLYMPYIEKVPLFKGCSPEFINQIAIRVHEEFFLPGEVILEQGNVVDQLYFICHGLL   459
Lactuca_GORK        KGSKTERYRDRMTDLLKYMNRNRLGRDIRNQIKGHLRLQYESNYTDSTILRDLPISIRAKISQTLYKSYIEKVSLFKGCSLEFINQVVTKVHEEFFLPGEVIMEQGNAVEQLYFICHGEL   451
Daucus_GORK         KGSKTERYRDKMTDIIKYMNRNRLGRDIRNQIKGHLRLQYESTYTDAAALQDLPISIRAKISQTLYMSCVENVSLFKGCSSEFISQIVTRVHEEFFLPGELIMEQGNVVDQLYIVCHGLL   457
Medicago_GORK       KGSKTEKFRDRMTDLMKYMNRNKLHKDIREQIKGHVRLQFESSYTDAAVLQDIPISIRSKISESLYSPYIKNVSLFSGCSSEFINQIVTRLHEEFFLPGEVIMQQGYVVDQLYFVCDGVL   456
Prosopis_GORK       KGSKTEKFRDRMADLLKYMNRNKLGRDIREQIKGHMRLQYESSYTEPAAIQDLPISIRSKISQDLYLPYIEKVPLFKTCSSEFINQIVTRLHEEFFLPGEVILEQGNVVDQLYFVCHGVL   464
Nicotiana_GORK      KGSKTERYRDKMTDLLKYMNRNRLGRDIRNQIKGHLRLQYESAYTDAAVLQDIPISIRAKISQSLYQSYIENVPLFKGCSSEFISQVVTRVHEEFFLPGEVIMEQGNVVDQLYFVCHGVL   439
Solanum_GORK        KGSKTERYRDKMTDLLKYMNRNRLGRDIRSQIKGHLRLQYESAYTDAAVLQDIPISIRAKISQNLYQSYIENVPLFKGCSLEFISQVVTRVHEEFFLPGEVIMEQGNVVDQLYFVCHGVL   447
Coffea_GORK         KGSKTEKYRDKMTDLIKYMNRNRLGKDIRNQIRGHLRLQYESSYTDAAVLQDIPISIRAKISQSLYKSYIEEVILFKGCSAEFINQIVTRVHEEFFLPGEVIMEQGHAVDQLYFVCHGVL   444
Sesamum_GORK        KGSKTERYRDKMTDLIKYMNRNRLGKNLRNQIKGHLRLQYESSYTDASVLQDIPISIRAKISQTLYKSYVENVPLFNGCSSEFINQIVTRVHEEFFLPGEVIMEQGNVVDQLYFVCHGVL   458
Cucumis_GORK        KGSKTVKFRDKMADVMKYMNRNRLSREIRDQIKGHLRLQYESSYTEATVLQDIPISIRAKISQTLYLPYVQNVSLFRGCSPEFINQIVIRLHEEFFLPGEVIMEQGNVVDQLYFVCHGVL   447
Vitis_GORK          KGSKTERFRDKMTDVIKYMNRNRLDRDVRNQIKGHLRLQYESGYTEASVIQDLPISIRAKIAQTLYKPLVEKVSLFRGCSLELINQIVIRVHEEFFLPGEVIMEQGNVVDQLYFVCHGML   438
Cannabis_GORK       KGSKTEKFRDKMTDLIKYMNRNRLGKDIRNQIKGHVRLQYESSYTEASALQDIPISIRAKISQSLYLPFMEKVSLFRGCSSEFINQIVVKVHEEFFLPGEVIMEQGNVVDQLYFVCHGSL   458
Gossypium_GORK      KGSKTEKFRDKMADVIKYMNRNKLDRDLRNQIKGHLRLQYESTYTEAAVLQDIPISIRAKISQSLYLPYIENASLFKECSSEFINQIVIRLHEEFFLPGEVIMEQGNVVDQLYFVCHGVL   449
                    ***.* ::**:* ::  :***::*  ::*.**  *:***::* :*:   ::*:* *:*::*:: **   :    *:  ** *:  *:  :::**:*:***:: :**  *:::*::. * :
```

```
                     β4             β5   β6  Helix P      β7        β8     Helix B       Helix C                                          ANK1
Arabidopsis GORK     EALVTKTDGSEESVTLLGPHTSFGDISIICNISQPFTVRVCELCHLLRLDKQSFSNILEIYFHDGRTILNNIMEEKE---SNDRIKKLESDIVIHIGKQEAELALKVNSAAFQGDFYQLK   548
Brassica_GORK        EALETKTDGTEDLVELLEPHTSFGDISIICNISQPFTIRVRSLCHLLRLDKQSFSNILEIYFHDGRKILNNLMEGKE---SNERIKKLESDIMIHIGKQEAELALKVNSAAFQGDIYQLK   547
Zingiber GORK        EGITIGEDGSEETLLLLEPNSSFGEISILCNIPQPYAVRVRELCRLLRLDKQLFANIMEIYFVDGRTILSNLIEEN-E--SNGRIKHLESDITFHIGKQEAALALQVNSAAYYGDLLQLK   572
Elaeis_GORK          EGVGIGEDGP----LELEPNSSFGEIAILCNIPQPFTVRVCELCRLLRIDKQIFTNILQIYFVDGRKILSNLLEGN-D---SIQIKQLEGDIIFHIGKQEAELALRVNNAAFYGDLHYLK   542
Phoenix GORK         EGVGIGEDGSKDTVLTLQPNSSFGEIAILCNIPQPFTVRVCELCRLLRIDKQVFTNILQIYFVDGRRILNNLLEGN-D---SNRIKQLESDITFHITKQEAELALRVNSDAFYGDLHHLK   545
Oryza_GORK           EGVGIGEDGQEETILMLEPESSFGEIAVLCNIPQPFTVRVCELCRLLRLDKQSFTNILEIFFVDGRRILSNLSESS-E--YGSRIKQLESDITFHIGKQEAELTLRVNNAAFYGDMHQLK   581
Brachypodium GORK    EGVGIGEDGQEETLLMLEPESSFGEIAILCNIPQPYSVRVCELCRLLRLDKQSFTNILEIYFVDGRKILSNLTENN-E--YGGRVKQLESDITFHIGKQEAELTLRVNNAAFYGDLHQLK   563
Panicum_GORK         EGVGIGEDGQEETLLMLEPESSFGEISILCNIPQPYTVRVCELCRLLRLDKQSFTNILEIYFVDGRRILSNLSE-S-E--YGGRVKQLESDITFHIGKQEAELILRVNSAAFYGDLHQLK   565
Papaver GORK         EEMGIGEEGSEVTVSLLEPNSSFGELSILCNMPQPYTIRVCELCRLLRLDKESFISILEICSSDERIILTNLLEGKESNKDFHRMKQLESDVMFHIGKQEGELALRVNSAACHGDLYHLK   571
Amborella_GORK       EEVGIGEDGLEETIVQLEHDNSFGEIAILCNIPQPYTVRVCELCRLLRLDKQSFSNILQIYFIDARTVLNNLLEGK-E--SSLRLKQLESDIIFHINKQEAELALRVNSAAYYGDLYHLK   551
Vulgaris GORK        EEVGIGQDGSEETVSRLEPGSTFGQISIFCNIPQPTTVRVLELCRLLRIDKESLSNIIDIYFYDGKKIFDNLLEGN-D--GKYNLKQVESDIASHIGKQESELALKVNNAAYHGDLYQIK   578
Chenopodium_GORK     EEVGIGQDGSEQIISRLEPNNTFGQISIFCNVPQPSIIRVIDLCRLLRIDKESLSNIIDIYFYDGKKIFDNLLEGNNN--GKYNLKQVESDIASHIAKQESELALKVNNAAHHGDLYQLK   577
Lactuca GORK         EEIGICEDGSEETVSILKPHSSFGEVSILCNIPQPYTVRVCELCRLLRLDKQSFSNILEIYFHDGRKILNNLVEFN-A--SDARMKQLETDIILHIGKHEAELALRVNSSAFYGDLYQLK   568
Daucus_GORK          EEVGICEDGTEETVSVLRPNSLFGEVSILCNIPQPYTVKVSELCRLLRLDKQSFSNILEIYFHDGRKILNNLLEGK-E--SNLRLKQLESDITFHINKQEAELALRVNSAAYYGDLYQLK   574
Medicago GORK        EEVGIADDGSEETVALLERNSSFGEISILCNIPQPHTVRVCELCRVLRIDKQSFSNILDIYFYDGKKVLDNLLEGK-E--S-IRGKQLESDITFHIGKLESELALKVNRTAFDGDLYQLK   572
Prosopis_GORK        EEVGPGENGSEETVSLLEPNSSFGQISILCNIPQPYTVRVCELCRLLRIDKQSFTDILDIYFYDGRKVLNNLLEGK-D--S-SRGKQLESDITFHIGKQEAELALKVNNAAFNGDLNHIK   580
Nicotiana GORK       EEVGIAKDGSEETVALLEPNSSFGDISIVCNIPQPYTVRVCELCRLIRIDKQSFSNILEIYFHDGRRILSNLLQGK-E--SNLRVKQLESDIAFHIGKHEAELALKVNSAAYHGDLHQLK   556
Solanum_GORK         EEVGIAKDGLEETVSLLEPNSSFGDISIVCNIPQPYTVRVCELCRLLRIDKQSFANILEIYFHDGRRILSNLLQGK-E--SNLRVKQLESDIALHIGKHEAELALKVNSAAYHGDLHQLK   564
Coffea GORK          EEVGTAKDGSEETLSLLEPNSVFGIVSILCNIPQPYTVRVCELCRLLRIDKQSFSNILEVYFHDGRTVLTNLLEEK-D--SAFSLKQLESDITLHIVKQEAELALKVNSSAAYYGDLQQLK   561
Sesamum_GORK         EEVGIAADGSEDTVSLLEPNSLFGEISILCNIPQPYTVRVCELCRLLRIDKQSFSNILEIYFHDGRKVLTNLLEVI-T--D-----STISDITFHIGKQEAELALKVNSAAYYGDLYQLK   570
Cucumis GORK         EELGIGQDSSEETIDLLQPNSSFGEISILCNIPQPYTVRVCELCRLLRIDKQSFTNILDIYFYDGRKILNNLLEGK-E--TNLRVKQLESDITFHIGKQEAELALKVNSAAYHGDLYQLK   564
Vitis_GORK           EEIGIGADGSEETVLPLQPNSSFGEISILCNIPQPYTVRVLELCRLLRIDKQSFTDILEIYFYDGRRILNNLLEGK-E--SNLRVKQLESDITFHIGRQEAELALRVNSASYHGDLYQLK   555
Cannabis GORK        EEVGIGEDGSEETVSLLQPSSSFGEISILCNIPQPYTVRVCELCRLLRIDKQSFTNILDIFFYDGRKILNNLLEVK-E--SNLRVKQLESDITFHIGKQEAELALKVNSSAFHGDLHQLK   575
Gossypium_GORK       EGIAIGEDGSEETVSLLEPNSSFGEISILCNIPQPYTVRVCELCRLLRIDKQSFSNILEIYFYDGRKVLNNLLEGK-E--SNLRVKQLESDISFHIGRQEAELALRVNCAAYNGDFYQLK   566
                     * :    :.       *   . ** :::.**: **  ::* .**:::*:**: : .*:::   * : :: *: :                  *:  ** : *. * *:**  :  **:  :*

                                             ANK2                                ANK3                          ANK4
Arabidopsis_GORK     SLIRSGADPNKTDYDGRSPLHLAACRGYEDITLFLIQEGVDVNLKDKFGHTPLFEAVKAGQEGVIGLLVKEGASFNLEDSGNFLCTTVAKGDSDFLKRLLSSGMNPNSEDYDHRTPLHVA   668
Brassica_GORK        SLVRSGADPNKTDYDGRAPLHLAASRGYEDITLFLIQEGVDINQKDKFGNTPLLEAVKAGQDRVIDLLVKEGASFDLEDAGNFLCTVVVKGDSDFLKRLLSSGMDPNTEDYDHRTPLHVA   667
Zingiber_GORK        GLVKAGADPKNTDYDGRSPLHLAASRGFEDITSFLIQECVDINVSDNFGNTPLFEAVKNGHDKVASLLYNNGAKLNLKDPSSHLCSAVARGDSDFIKRLLAYGIDLNLKDYDSRTPLHVA   692
Elaeis_GORK          GLIQAGADPNKTDYDGRSPLHLAAAKGHEDITLFLIQEGVNINLSDNFGNTPLFEAVKVGHDRVASLLFSRGAQLNLKDAGSHLCTAAAKGDSDFIRRALSYGVDPNSKDYDHRTALHIA   662
Phoenix_GORK         GLIQAGADPNKTDYDGRSPLHLAAARGHEDITLFLIQEGVDINLSDNFGNTPLFEAIKDGHDRVASLLFSRGAQLSLKDAGSQLCAAAAEGDSDFIKRALSYGVDPNSKDYDHRTALHIA   665
Oryza_GORK           SLIRAGADPKNTDYDGRSPLHLAACKGFEDVVQFLLHEGVDIDLSDKFGNTPLLEAVKQGHDRVATLLFSKGAKLSLENAGSHLCTAVARGDTDFVRRALAYGGDPNARDYDHRAPLHIA   701
Brachypodium_GORK    GLIRAGADPKNTDYDGRSPLHLAASKGYEDVAQFLIHEGADINLIDKFGNTPLLEAVKQGHDRVATLLFKKGAILNLQNAGSHLCSAVSKGDSDFIRRALACGADPDSKDYDHRSPLHIA   683
Panicum_GORK         SLIRAGADPKNTDYDGRTPLHLAACRGYEDVVQFLLAEGVDINLTDQFGNTPLLEAVKQGHERVAALLFAKGAKLSLKNAGSHLCTAVAKGDSDFIRRALAFGADPNCGDYDHRTPLHIA   685
Papaver_GORK         RLIRAGADPNKTDYDGRTPLHIAASEGFEHIASFLIQEGVGVNIADKFGNTPLFEAVKRGNDRVASLLVKEGASLNIVDGGSCLCQAVSRGDSDFVKRLLSNGLDPNSKDYDQRTALHVS   691
Amborella_GORK       GLIRAGADPKKTDYDGRSALHLAASKGFEDITTFLIRETVDVNLTDKFGNTPLLEAIRYGHDRVASLLVKEGASLDIDDAGGFLCKAVASGNTDFLKRLLVYGMDPNSRDYDYRTALHKA   671
Vulgaris_GORK        NLLNAGADMNKTDYNGRSPLHIAAARGCEEIILLLVKEGVRINEKDNFGNTPLLEAVKNGNDKAAALLYREGARLDVKDVGSFLCTAVLGGDVDYLRRILANGIDPNSKDYDYRTPLHVA   698
Chenopodium_GORK     SLLNAGADLKKTDYNGRTPLHIAASGGCEDIVLFLIEEGADVNRTDNFGNTPLLEAVKNGHDKVAALLYKEKARIDVKDVGSFLCTVVLSGDVEFLKRILTNGMDPNSKDYDHRTPLHVA   697
Lactuca_GORK         SLIRAGADPNKKDYDGRTPLHLAASKGYEDITLFLIQEKVEVNVLDNFGNTPLLEAIKNGHDKVASILVKEGGSLKIDDAGWFLCTSVARGDIDYIRRILSNGVDPNSKDYDFRTPLHVA   688
Daucus_GORK          GLVRAGADPNKTDYDGRSPLHLAASKGYEDIILFLTQEGVDINISDNFGNTPLFEAIKSGHDQVVSLLIKEGAILKIGDSGPFLCTAVARGDYDLIKRVLSAGIDPNSSDYDHRTPLHVA   694
Medicago_GORK        SLIRAGADPKKTDYDGRSPLHLAACRGYEDITLLLIHERVDMNVKDNFGNTPLLEAVKNGHDRIASLLVKEGASLKIDDGGSFLCSAVARGDSDYLKRLLSNGMDANLKDYDYRTPLHVA   692
Prosopis_GORK        GLIRAGADPNKADYDGRSALHLAASRGFEDITIFLIQEGVEIDLRDNFGNTPLLEAVKNGHDRVASLLVGKGASMKIEDAGSVLCNAVARGDSDYLKRLLSYGMDPNTKDYDYRSPLHVA   700
Nicotiana_GORK       SLIRAGADPNKKDYDGRSPLHLAASRGYEDITLFLIQEGVDINAPDKFGNTPLLEAIKSGHDRVASLLVKEGAFLKIENAGSFLCMVIAKGDSDLLRRLLSNGVDPNSKDYDQRTPLHVA   676
Solanum_GORK         SLIRAGADPKKKDYDGRSPLHLAASRGYEDITLFLIQEGIDINAPDKFGNTPLLEAIKIGHDRVASLLVKEGALLNIENAGSFLCMVIARGDSDLLRRLLSNGVDPNTKDYDQRTPLHVA   684
Coffea_GORK          NLIRAGADPNKNDYDGRSPLHLAASRGYEDIAQFLIQEGVDINVTDNFGNTPLLEAIKSGHEHVASLLVKEGASLQIENAGNFLCTVVAKGDSDLLRRFLTRGIDPNSKDYDQRTPLHVA   681
Sesamum_GORK         SLIRSGADPNKKDYDGRSALHLAASRGYEDVTLFLIQEGVDINAEDNFGNTPLLEAIKSGHDRVASLLSREGALLKIDNPGAYLCTVVSKGDTDLLRRVLANGIDPNSKDYDHRTPLHLA   690
Cucumis_GORK         GLVRAGADPNKTDYDGRSPLHLAASRGFEDIVVFLIQEGVNIDLKDNFGNTPLMEAIKNGNDQVAVLLSKEGASLKVDNPGSFLCTAVSRGDSDLVKRLLCYGIDPNSKDYDSRTPLHIA   684
Vitis_GORK           SLIRAGADPNKTDYDGRSPLHLASARGFEDIVTFLIQEGVDVNISDNFGNTPLLEAIKNAHDRVASLLVNKGALLKIDDAGGFLCATIARGDSDFLKRILSNGIDPNSKDYDHRTPLHVA   675
Cannabis_GORK        SLIRAGADLNKTDYDGRSPLHLAASRGYEDITRFLIQQGVNVNAIDNFGNTPLLEALKNGHDRVSSLLVQEGASLMIDNAGSFLCTAVSRGDSDFLKRVLSNGVDPNSKDYDHRSPLHVA   695
Gossypium_GORK       SLIRAGADPNKTDYDGRSPLHLAASKGYEDITSFLIRHPVDINLKDKFGNTPLLEAIKNGHDNLAALLIKEGASLNIDDAGSYLCTAVAKGDSDFLRRLLSNGVDPNSRDYDHRTPLHVA   686
                      *:.:*** :: **:**: **:*:. * *.:  :*  .   ::  *:**:***:**:: .::    :*  . . : : : .  **     *: : ::* *  * : :  *** *: ** :
```

```
                          ANK5                              ANK6                                                                                  KHA

Arabidopsis_GORK    ASEGLFLMAKMLVEAGASVISKDRWGNSPLDEARLCGNKKLIKLLEDVKNAQSSIYPSSLRE-------LQ-EERIERRKCTVFPFHPQEAK----EERSRKHGVVVWIPSNLEKLIVTA   776
Brassica GORK       ASEGLFLMAKMLVEAGASVVAKDRWGNSPLDEARMCGNKKLIKLLEDADTSQPYIRPSSFHE-------PQ-DEKFERRKCTVFPFHPHE-------EPSRKHGVMVWLPRDLQKLVETA   772
Zingiber_GORK       ASVGFYAIAKMLSEAGASVFSVDRWGNTPLDEAMKCGSESMVMLLEDAKSQELSKFPERAQE-------LQ-EKMQSRRRCTVFPYHPRDL-----IESR-KEGVMLWIPHTLEELINSS   798
Elaeis GORK         ASEGLYFIAKLLLEAGASVFAMDRWGATPLDEGRKSGNQSLMLLLEGAKSDELSKFPEHARE-------VQ-DKMHP-RRCTVFPFHPWDP-----KEAR-REGVVLWIPCTIEELIRSS   767
Phoenix_GORK        AAEGLYFIAKLLLDAGASVFAMDRWGATPLDEARKSGNKSLMLLLEGAKSDELSKFPERARE-------VQ-DKMHP-RRCTVFPFHPWDS-----KEER-REGVMLWIPCTMEKLIRSS   770
Oryza GORK          AAEGLYLMAKLLVDAGASVFATDRWGTTPLDEGRRCGSRTMVQLLEAAKSGELSRYPERGEE-------VR-DKMHP-RRCSVFPHHPWDG-----GERR-REGVVVWIPHTIEGLVSSA   806
Brachypodium_GORK   AAEGLYMMAKLLVEAGASVFATDRWGTTPLDEGRKSGSKPLMMLLEQAKAEELSKFPARSEE-------VR-DKMHP-RRCSVFPNHPWDT-----DGKR-KEGVTLWIPHTIDWLIRSA   788
Panicum GORK        AAEGLYLIAQMLVEAGASVFATDRWGTTPLDEARKCGGRTLLALLEQARAEELSKFPERGDE-------VR-DKMHP-RRCSVFPYHPWRAAAGTGAGRR-KEGVVLWIPHTIEGLVASA   795
Papaver_GORK        VSQGSYMIAKVLIEGGASVLTRDRWGNTPVDEGRTSGNKKLIKLLEEAESAELSTASTSGSAIDNTSELQVTNKANP-RKCTVFPFHPWDP-----KEKR-KEGVVLWVPPSVDELISMA   804
Amborella GORK      AAEGLYSLAKNLLEAGASVFSKDRWGNTPLDEGHRSGSKLVINLLEEAKIDQLSRYPNYSQE-------IQ-GKIEA-KKCTVFPFHPWGP-----REER-KEGVVMWIPPTLDQLLSSS   776
Vulgaris_GORK       CSQGLYLMAKVLVEAGAYVTLKDRWGNTALDEAWMCGNKNLIKLLETAKSAQLSQSSGNIEE-------LS-DKKLQ-KKCTVFPFNPWGS-----KENR-RPGIVLWVPRTIDDLAKLA   803
Chenopodium GORK    CSQGLYLMAKLLVEAGAYVTLKDRWGNTPLDEAWMCGNKNLIKLLEAAKAAQLAQSSDHIEE-------ML-DKKLH-KKCTVFPFHPWGT-----KENR-KPGIVLWLPRTIDELIRLA   802
Lactuca_GORK        ASQGSYIIVKLLVEAGASVLSKDRWGNTPLDEGRMSGNKMLIKLLEEAKDVQLSELPEGSQE-------IT-DKMHP-RKCTVYAFHPWEE-----KDKI-TCGVVLWIPRTTEQLIKTA   793
Daucus GORK         ASQGLYLLAKLLLESGASVISRDRWGNTPLDEGRMSGNKNLIKLLEIAKSSQLAEISNQSPE-------IT-DKPQP-KKCRVYPFHPDKP-----KENKPSYGVMLWVPETMEELIKKA   800
Medicago_GORK       ASEGLIFMAKLLLEAGASVFTKDRWGNTPLDEARMSGNKNLIKLLEDAKSAQLTEFPF-PQE-------IT-DKVHP-KKCTVFPFHPWDP-----KEQR-RNGIVLWIPHTIQELIITA   796
Prosopis GORK       AAEGLFLMAKLLIEAGASVFIQDRWGNTPLDEARMCGNKNLIELLEDARSAQLSRFPHLSSE-------LT-VKLHP-KKCTVFPFHPWGP-----KEHR-RHGIVLWVPHTIEELIETA   805
Nicotiana_GORK      ASQGLYSMAKLLLGAGASVFTKDRWGNTPVDEARVSGNKQMIGLLEEAKSAQLSEFPDVPHE-------IS-EKLRP-RKCTVFPFHPWES-----KDVR-KHGVVMWIPQTIEELVTTA   781
Solanum GORK        ASQGQYSMAKLLLGAGASVFSKDRWGNTPVDEARVSGNKQMISLLEEAKSAQLCEFPDVPHE-------IS-DKLRP-RKCTVLPFHPWES-----KDLR-KHGVVLWIPQTIEELVTTA   789
Coffea_GORK         ASQGQYFMAKLLLDAGASVFARDRWGNTPLDEGRISGNKNMMDLLEEAESAQSSELSDLSQE-------STADKMLR-RKCTVFPFHPWEP-----KDAR-KCGVVLWVPDSIEELIREA   787
Sesamum GORK        ASQGLYLMAKLLVEAGASVFSKDRWGNTPLDEGRLCGNKNMIRLLEEAKKAQLSEYPECSQE-------VT-DRKHT-KKCTVFPFHPWDP-----KEGR-KHGIVMWVPNTIDELIKAA   795
Cucumis_GORK        VSEGLTLMAKLLLESGASVFSKDRWGNTPLDEGRICGNKNMLKLLEEAKASQLSESPYSSRE-------FT-DKKPT-KKCTVFPFHPWDP-----EENK-RPGIMLWVPLTIEELIKES   789
Vitis GORK          ASEGLYFMAKLLLEARASVFSKDRWGNTPLDEGWKCGNKNLMKLLEDAKVAQLSEFPDCSRE-------IT-DKMHP-RKCTVFPFHPWDP-----KEHK-RPGIMLWVPQTIEELIKTA   780
Cannabis_GORK       ASEGLYLMAKLLIEAGANVFSKDRWGNTPLDEGRMCGNKNLIKLLEDAKTAQLLDFPNSGRE-------IT-EKIHR-RKCTVFPFHPWDP-----KELR-RPGIVLWVPNSIEQLINTA   800
Gossypium GORK      ASEGLYIMAKLLIEAGASVFSKDRWGNTPLDEARMCGNKNLIKLLEDAKSTQLSELPHCSKE-------FT-DKIHP-KKCTVFPFHPWDA-----KDQR-RHGIVLWVPHTMEALVTTA   791
                     : *   :.: *  . * *   **** : :**.  .*.. :: *** .   :                      .    ::* *   :*         *: :*:*   : *   :

Arabidopsis_GORK    AKELGLSDG-ASFVLLSEDQGRITDIDMISDGHKLYMISDTTDQT-------   820
Brassica GORK       AQELGISNE-VPFVILSEEGGRITDIDMISDGQKLYLISDSTDQST------   817
Zingiber_GORK       QELLNCSVS----RLLSEEGGRILPAGLITDNLAPSVHAQNTTSTSDKELN-   845
Elaeis_GORK         REQLKSSGS----CILSEDGGRILDVDMISDGQKLYLVADQETTAHNNQHT-   814
Phoenix_GORK        REQLKSSGS----RILSEDGARILDVDMISDGQKLYLVADQETSTHNNQHT-   817
Oryza_GORK          QEKLGLAGSGEGLRLLGEDGARVLDVDMVHDGQKLYLVVGGGGDDGGTEARQ   858
Brachypodium_GORK   QEKLGLSGS--CLRLLGEDGARVQDVDMVNDGQKLYLVGDEDVGRSE-----   833
Panicum_GORK        QEKLGVPGPGARLRLLCEDGARVLDVDMVNDGQKLYLVGGDDDDQEDGE---   844
Papaver_GORK        SEHLQFYD--GS-RILSEDGGKILYIEMIHDGQKLHLV-------------   839
Amborella_GORK      SELLGCF---CT-CILSEDAGKILSVDMISDGQKLYAISDDSHIRDSL----   820
Vulgaris_GORK       SEKLNFPS--GS-CILSDDGGKILDIDMIENGQKLYLISETE----------   842
Chenopodium_GORK    SEKLNFLT--GS-CILSEDGGKILDVDMIENGQKLYLISETE----------   841
Lactuca_GORK        AEQLKLENYDTC-CIVTEDAGKILDVDMILDGQKLYLISEE-----------   833
Daucus_GORK         AEQLNCPQ--NS-CILSEDAAKILDADMISDGQKLYLIDETQ----------   839
Medicago_GORK       AEQIGFSS--DA-CILSEDAGKIIDISMIKDDQKLYLVNETH----------   835
Prosopis_GORK       AEQLNVSS--DC-SILSEDAGKITDANLIMDGQKLHLVHETH----------   844
Nicotiana_GORK      SEKLNFPS-D-S-CILSEDAGKILDIDMIADGQKLYLINDQST---------   821
Solanum_GORK        SEQLDFPS-GTS-CILSEDAGKILDVDMIVDGQKLYLINEST----------   829
Coffea_GORK         SKQLNFAS--CT-CILSEDAGKIIDVDMISNNQKLYLMNETD----------   826
Sesamum_GORK        SDQLESPN--GS-CILSEDGGKIFDVDMITDGQRLYMINETD----------   834
Cucumis_GORK        SEQLQVSG--EC-CILSEDGGKILDVHMIDESQKLYLVPDTH----------   828
Vitis_GORK          TEGLQFSS--ES-CILSEDGGKILDVDMISDGQKLYLLCETLDI--------   821
Cannabis_GORK       ADQLEISS--AA-CILSEDGGKILEEDMINDGQKLYLVGDTYSA--------   841
Gossypium_GORK      AEQLDLAG--AS-CMLTENGGKILDVDMINDGEKLYLIGGTH----------   830
                     . :          :: :: .::    :: :.
```

The cryo-EM structure of *At*GORK is used to restrict sequence gaps to inter-helical segments, with the superior coils and arrows defining extents of the secondary elements. The sequences include representative members from dicots and monocots: Arabidopsis thaliana (NP_198566.2), Brassica rapa (XP_009102317.1), Zingiber officinale (XP_042469382.1), Elaeis guineensis (XP_010905454.2), Phoenix dactylifera (XP_008795354.1), Oryza sativa Japonica Group (NP_001408448.1), Brachypodium distachyon (XP_003560852.1), Panicum virgatum (XP_039805885.1), Papaver somniferum (XP_026377173.1), Amborella trichopoda (XP_011629401.1), Beta vulgaris subsp. vulgaris (XP_010693307.2), Chenopodium quinoa (XP_021754949.1), Lactuca sativa (XP_023769988.1), Daucus carota subsp. sativus (XP_017218838.1), Medicago truncatula (XP_003616247.2), Prosopis alba (XP_028768743.1), Nicotiana tabacum (XP_016460239.1), Solanum lycopersicum (XP_004250206.1), Coffea arabica (XP_027077850.1), Sesamum indicum (XP_020551503.1), Cucumis sativus (XP_004140369.2), Vitis vinifera (XP_010660282.1), Cannabis sativa (XP_030505721.2), Gossypium hirsutum (XP_016749823.2).

Figure S3. Sequence alignments for the representative GORKs from both dicots and monocots.

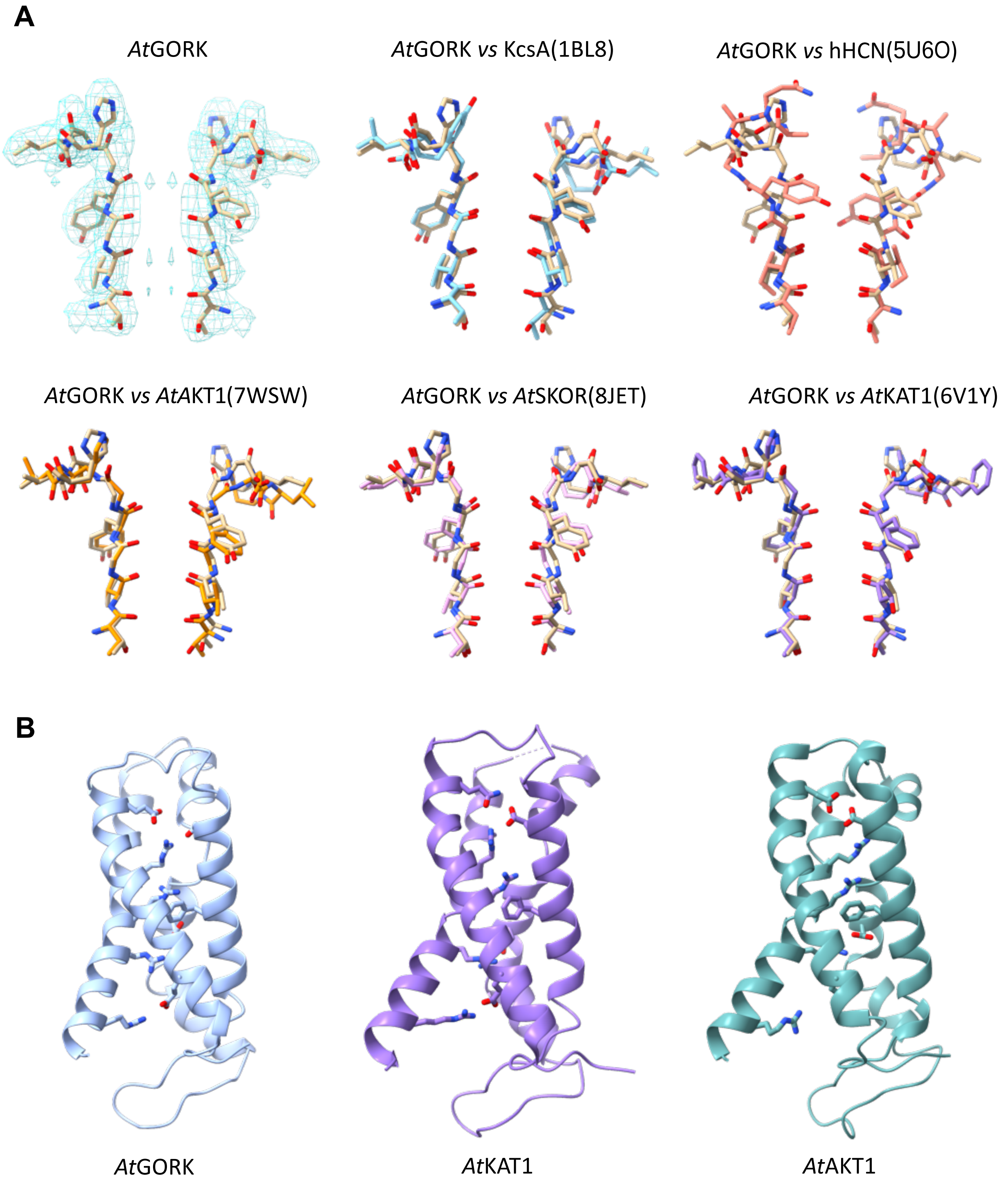

Figure S4. Structural comparison of K^{+}-selectivity filter and VSD of *At*GORK with other K^{+} channels.

(**A**) Comparison of K^{+}-selectivity filters from *At*GORK with other K^{+} channels. (**B**) Comparisons of voltage sensing domain (S1-S4) from *At*GORK (light-blue), *At*KAT1(purple, 6V1Y) and *At*AKT1 (dark-green, 8WSW). The S4 helix in these three K^{+}chan nelcontains conserved positively charged residues, as shown in sticks, and adopt a resting "up" conformation.

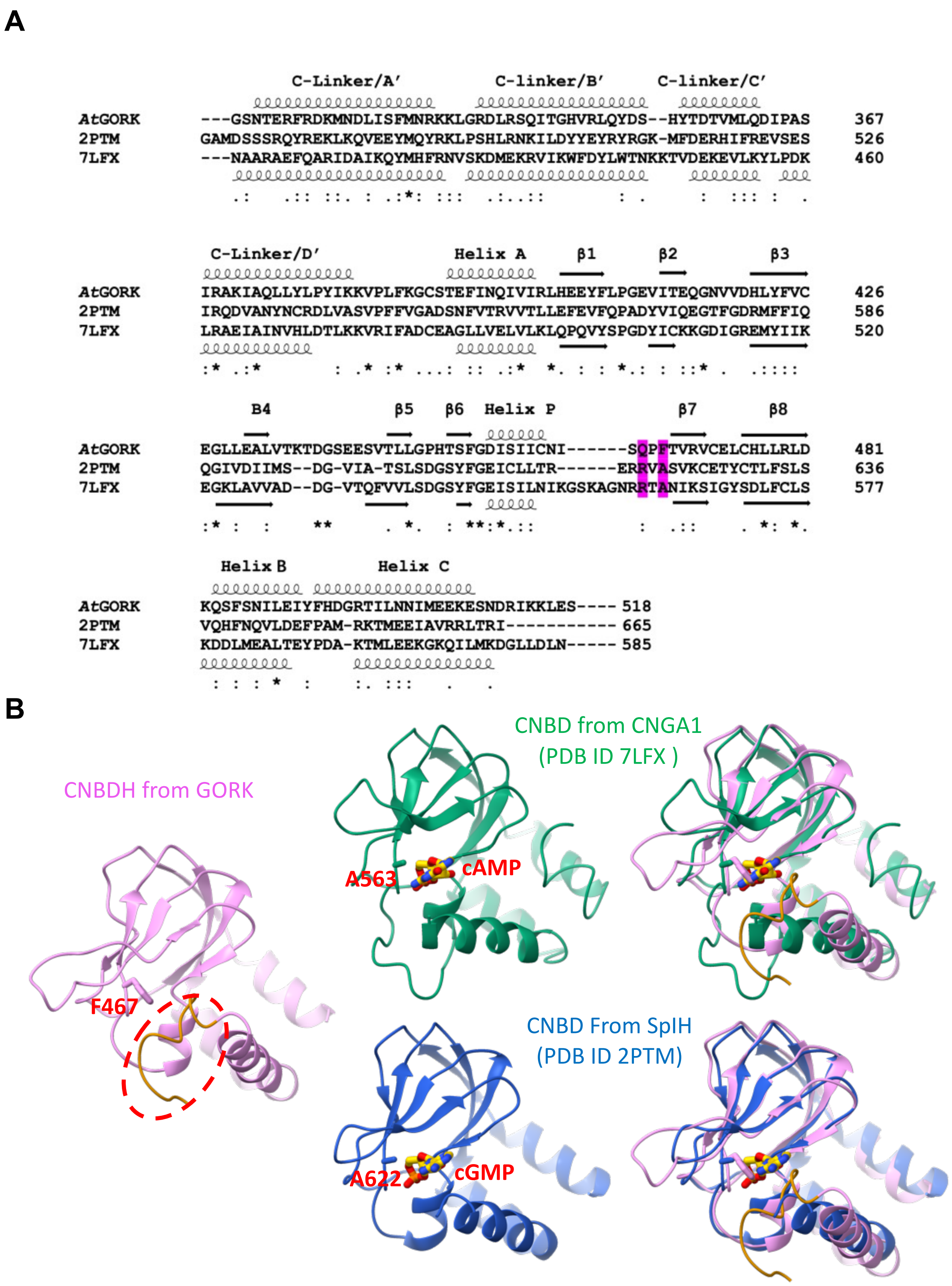

Figure S5. Structural comparison of CNBD domain of *At*GORK with other K$^+$ channels.

(**A**) Structure-based sequence alignment of CNBDH of *At*GORK with CNBD from other K$^+$ channels. (**B**) Comparisons of CNBDH of *At*GORK with CNBDs from CNGA1 (7LFX, green) and SpIH (2PTM, blue). A small side-chain residue alanine (A 563in 7LFX, or A622 in 2PTM), located at the entrance of the cAMP/cGMP binding pocket, is replaced by bulky phenylalanin e (F467in *At*GORK), which likely blocks access for the secondary messenger molecules. Helix C adopts a closed conformation, with itsadjacent connecting loop (red circle) to the next domain positioned at the entrance of the cAMP/cGMP binding site, in dicatingthat large conformational changes would be required for cAMP/cGMP binding.

A

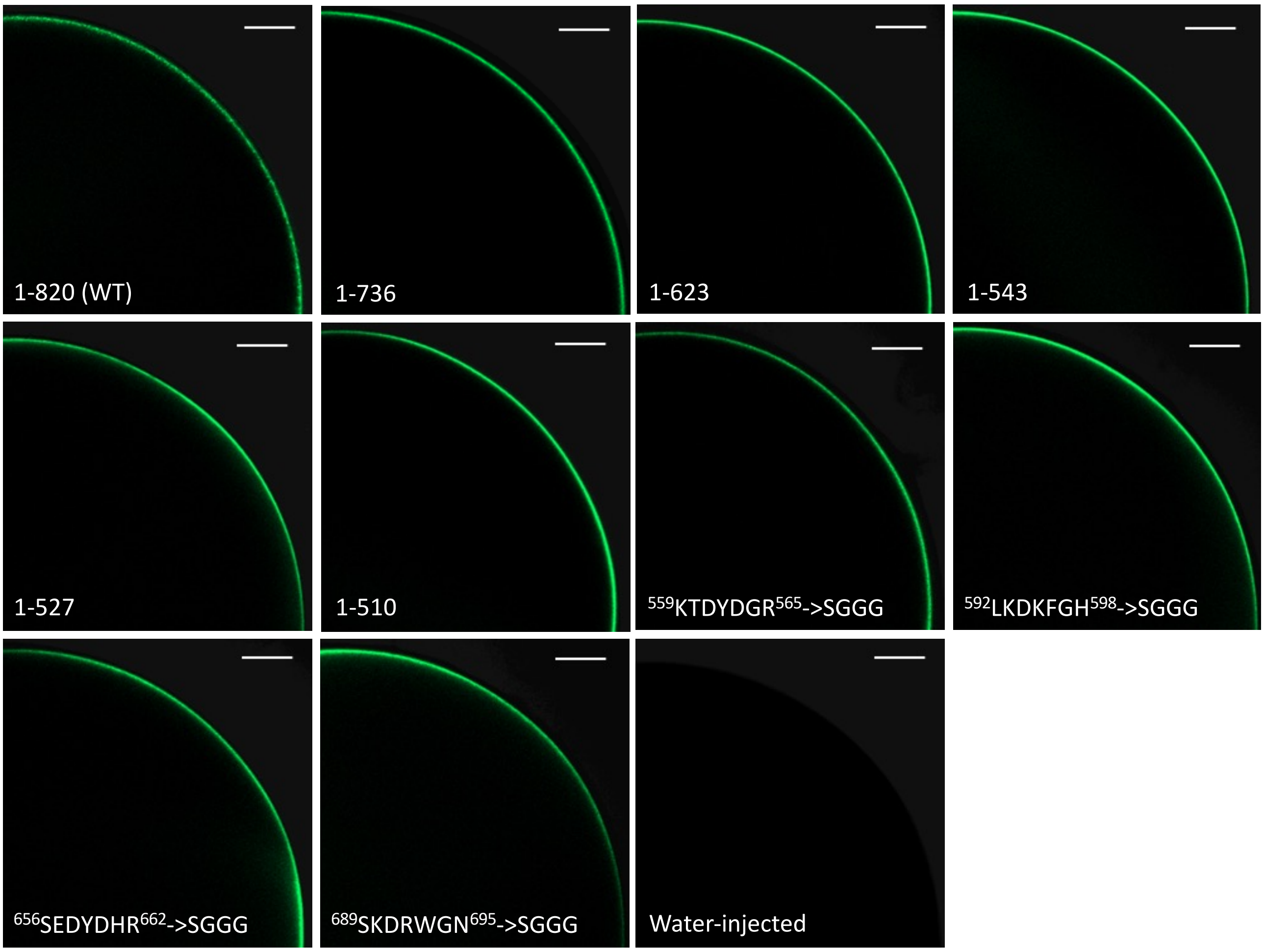

B

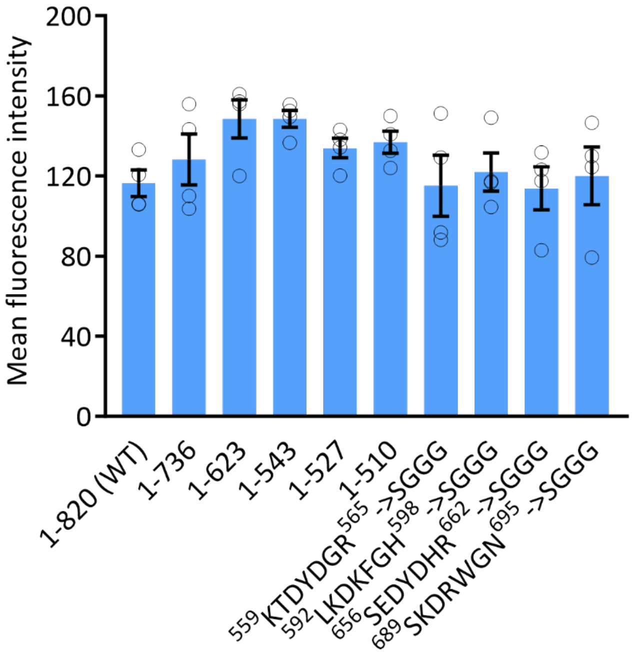

Figure S6. Fluorescence and confocal imaging of the N-terminal GFP tagged *At*GORK.

(**A**) Representative fluorescence images of *At*GORK wild-type (1-820) and mutants corresponding to Figure 3. The GFP fluorescence on the plasma membrane of oocytes was analyzed by LSM980 laser confocal microscope. The quarter of the whole cell is shown. Scale bars: 100 μm. (**B**) Mean fluorescence intensity measurements in ImageJ. A one-way ANOVA analysis was performed with a P value of 0.1298. Data are mean ± SEM, $n = 4$.

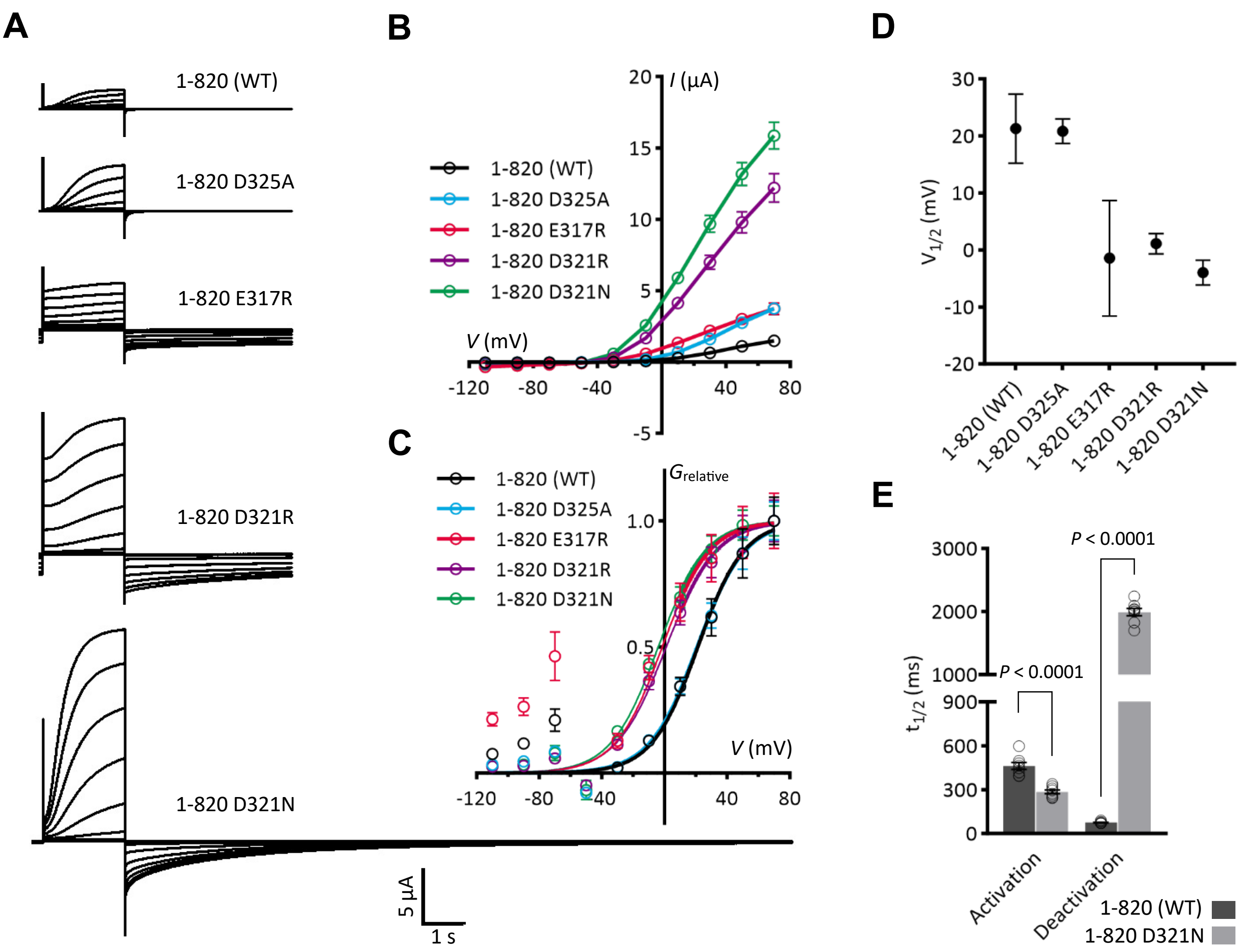

Figure S7. Mutational tests of conserved acidic residues at the C-linker/TMD interface in full-length *At*GORK.

(**A**-**E**) Electrophysiological analyses of E317, D321 and D325 mutations in *At*GORK$^{1\text{-}820}$. Representative current traces (**A**) and steady-state current-voltage (*I*-*V*) relations (**B**) are shown. Relative conductance-voltage ($G_{relative}$-V) curves (**C**) and half-activation voltage ($V_{1/2}$) values (**D**) were generated through Boltzmann sigmoidal fitting (outliers excluded). Conductance was calculated using the equation $G = I/(V - E_K)$, where I is the steady-state current, V is the test potential, and E_K (–58 mV) was derived from the Nernst equation based on intracellular (~100 mM) and extracellular (10 mM) K^+ concentration in the oocyte TEVC recordings. Relative conductance was calculated by normalization to the maximal conductance. Halftime ($t_{1/2}$) for activation at +70 mV and deactivation at −110 mV, calculated as ln(2)·τ where τ is the time constant from single-exponential decay fitting, is shown in (**E**). Data are mean ± SEM, $n \geq 8$. Significance analysis was performed using unpaired Student's t-test, with *P*-values displayed on the bar charts.

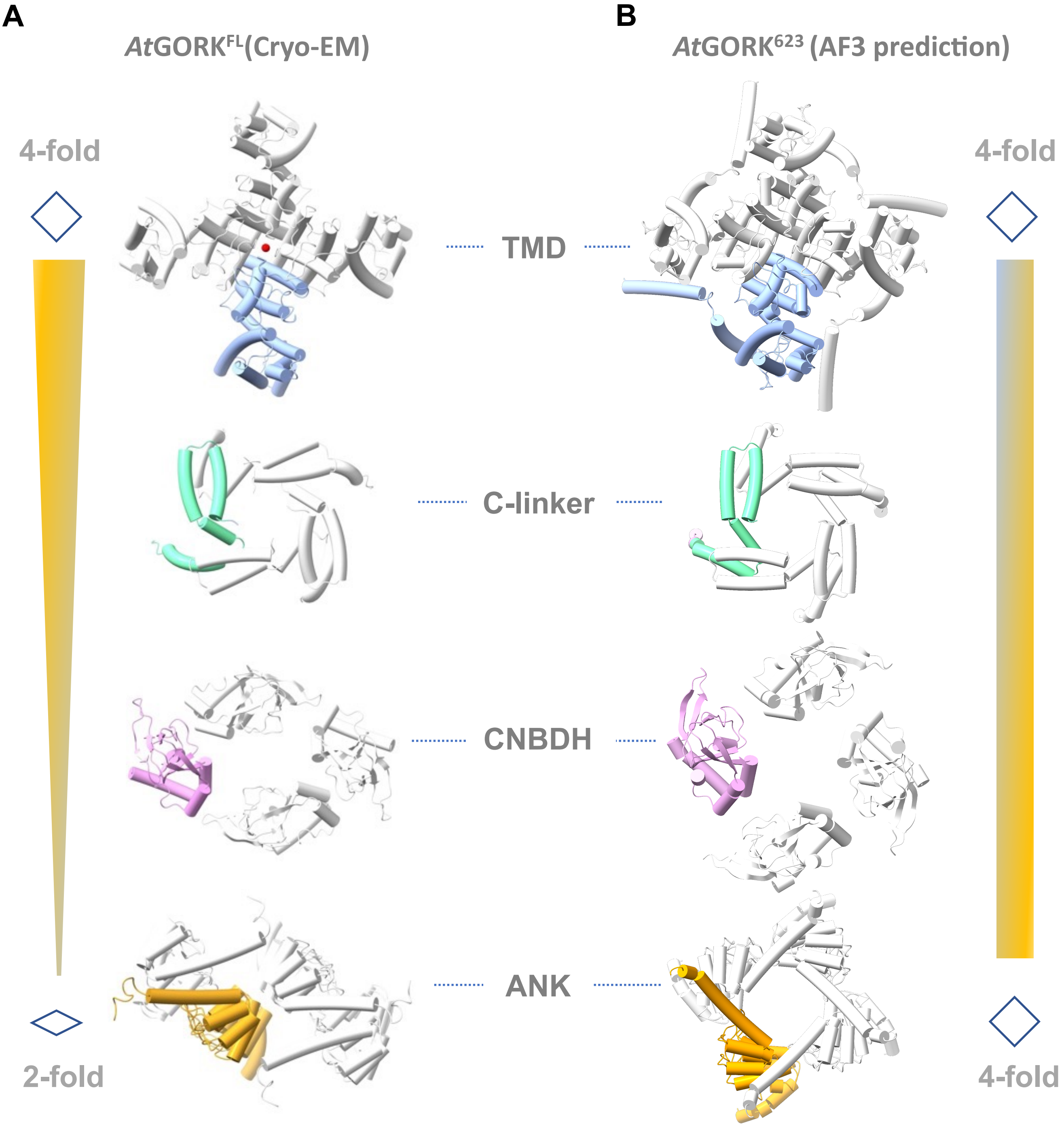

Figure S8. Symmetry analysis of domain assembly in *At*GORK^{FL}(Cryo-EM) and *At*GORK^{623}(AF3 prediction).

Table S1: Statistics of data collection, image processing, and model building

**Sample**	AtGORK^{FL1}	AtGORK^{FL2}	AtGORK^{623}	AtGORK^{510}
**PDB**	9KHF	8WFZ	9KHE	9KHG
**EMDB**	62338	37500	62337	62339
**Data collection and processing**				
Microscope	Titan Krios	Titan Krios	Titan Krios	Titan Krios
Detector	Gatan K3	Gatan K3	Gatan K2	Gatan K3
Magnification	22,500 ×	22,500 ×	130,000 ×	22,500 ×
Voltage (kV)	300	300	300	300
Electron exposure (e-/Å^2)	50	50	50	50
Nominal defocus range (µm)	-1.2 to -2.0	-1.2 to -2.0	-1.2 to -2.0	-1.2 to -2.0
Frames per movie	32	32	32	32
Pixel size (Å)	1.06	1.06	1.04	1.06
**Reconstruction**				
Software	cryoSPARC 3.2	cryoSPARC 3.2 & Relion 3.0	cryoSPARC 3.2	cryoSPARC 3.2
Symmetry imposed	C2	C1	C4	C4
Initial particle images (no.)	18,650,563	18,650,563	1,139,470	2,840,921
Final particle images (no.)	156,313	39,551	75,626	141,755
Map resolution (Å)	3.4	4.3	3.2	3.3
**Refinement and model validation**				
CC (mask)	0.7	0.76	0.82	0.84
CC (box)	0.63	0.66	0.58	0.64
CC (peaks)	0.55	0.45	0.54	0.62
CC (volume)	0.66	0.76	0.81	0.80
RMSD Bond Length (Å)	0.003	0.003	0.003	0.003
RMSD Bond angles (degrees)	0.632	0.701	0.627	0.572
Favored (%)	94.96	96.52	95.47	97.83
Allowed (%)	4.85	3.48	4.53	2.17
Ramachandran plot outliers (%)	0.19	0.00	0.00	0.00
Molprobity score	2.01	1.97	1.91	1.57
Clash score	14.33	17.44	11.95	10.17

Reference

Deng, Y.N., Kashtoh, H., Wang, Q., Zhen, G.X., Li, Q.Y., Tang, L.H., Gao, H.L., Zhang, C.R., Qin, L., Su, M.*, et al.* Structure and activity of SLAC1 channels for stomatal signaling in leaves. Proc Natl Acad Sci U S A 2021;**118**.

Emsley, P., Lohkamp, B., Scott, W.G., and Cowtan, K. Features and development of Coot. Acta Crystallogr D Biol Crystallogr 2010;**66**:486-501.

Liebschner, D., Afonine, P.V., Baker, M.L., Bunkóczi, G., Chen, V.B., Croll, T.I., Hintze, B., Hung, L.W., Jain, S., McCoy, A.J.*, et al.* Macromolecular structure determination using X-rays, neutrons and electrons: recent developments in Phenix. Acta Crystallogr D Struct Biol 2019;**75**:861-877.

Mastronarde, D.N. Automated electron microscope tomography using robust prediction of specimen movements. J Struct Biol 2005;**152**:36-51.

Pettersen, E.F., Goddard, T.D., Huang, C.C., Couch, G.S., Greenblatt, D.M., Meng, E.C., and Ferrin, T.E. UCSF Chimera--a visualization system for exploratory research and analysis. J Comput Chem 2004;**25**:1605-1612.

Pettersen, E.F., Goddard, T.D., Huang, C.C., Meng, E.C., Couch, G.S., Croll, T.I., Morris, J.H., and Ferrin, T.E. UCSF ChimeraX: Structure visualization for researchers, educators, and developers. Protein Sci 2021;**30**:70-82.

Punjani, A., Rubinstein, J.L., Fleet, D.J., and Brubaker, M.A. cryoSPARC: algorithms for rapid unsupervised cryo-EM structure determination. Nat Methods 2017;**14**:290-296.

Wu, C., Huang, X., Cheng, J., Zhu, D., and Zhang, X. High-quality, high-throughput cryo-electron microscopy data collection via beam tilt and astigmatism-free beam-image shift. J Struct Biol 2019;**208**:107396.

Zivanov, J., Nakane, T., Forsberg, B.O., Kimanius, D., Hagen, W.J., Lindahl, E., and Scheres, S.H. New tools for automated high-resolution cryo-EM structure determination in RELION-3. Elife 2018;**7**.
